# Surface Chemistry Dictates the Enhancement of Luminescence and Stability of InP QDs upon c-ALD ZnO Hybrid Shell Growth

**DOI:** 10.1021/jacsau.3c00457

**Published:** 2023-11-01

**Authors:** Ona Segura Lecina, Mark A. Newton, Philippe B. Green, Petru P. Albertini, Jari Leemans, Kenneth P. Marshall, Dragos Stoian, Anna Loiudice, Raffaella Buonsanti

**Affiliations:** † Laboratory of Nanochemistry for Energy (LNCE), Institute of Chemical Sciences and Engineering (ISIC), École Polytechnique Fédérale de Lausanne, CH-1950 Sion, Switzerland; ‡ The Swiss-Norwegian Beamlines, European Synchrotron Radiation Facility (ESRF), 38000 Grenoble, France

**Keywords:** quantum dots, oxides, hybrids, core−shell, ligands

## Abstract

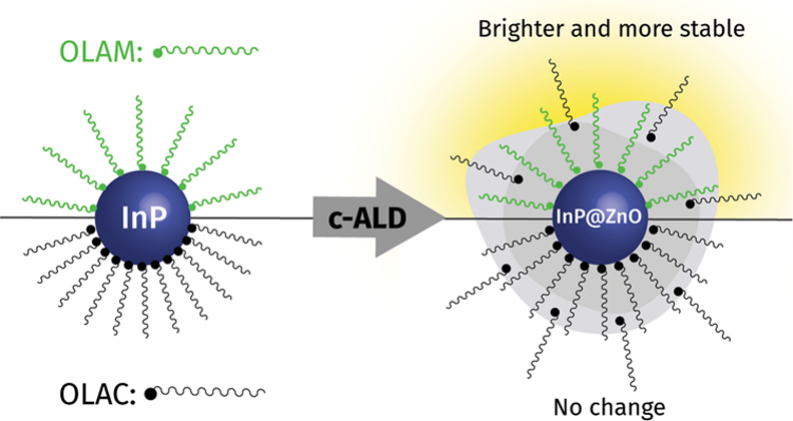

Indium phosphide quantum dots (InP QDs) are a promising example of Restriction of Hazardous Substances directive (RoHS)-compliant light-emitting materials. However, they suffer from low quantum yield and instability upon processing under ambient conditions. Colloidal atomic layer deposition (c-ALD) has been recently proposed as a methodology to grow hybrid materials including QDs and organic/inorganic oxide shells, which possess new functions compared to those of the as-synthesized QDs. Here, we demonstrate that ZnO shells can be grown on InP QDs obtained via two synthetic routes, which are the classical sylilphosphine-based route and the more recently developed aminophosphine-based one. We find that the ZnO shell increases the photoluminescence emission only in the case of aminophosphine-based InP QDs. We rationalize this result with the different chemistry involved in the nucleation step of the shell and the resulting surface defect passivation. Furthermore, we demonstrate that the ZnO shell prevents degradation of the InP QD suspension under ambient conditions by avoiding moisture-induced displacement of the ligands from their surface. Overall, this study proposes c-ALD as a methodology for the synthesis of alternative InP-based core@shell QDs and provides insight into the surface chemistry that results in both enhanced photoluminescence and stability required for application in optoelectronic devices and bioimaging.

## Introduction

Colloidal quantum dots (QDs) are solution-processed nanocrystals of semiconductor materials. Since their discovery more than four decades ago, the interest in QDs has grown continuously because of their widely tunable size-dependent properties and solution processability.^1−3^

Hybrid materials incorporating QDs and organic molecules, such as photoactive chromophores, are important for advanced applications in photocatalysis, solar energy harvesting, and bioimaging.^4−6^ Established chemical strategies based on silica or polymer coatings on QDs enable the assembly of such hybrid materials as a single unit.^7−12^ For instance, silica shells have been used to confer stability in aqueous media to luminescent QDs and allowed their functionalization with antibodies for biolabeling.^12^ In another example, amphiphilic polymers have been used to trap photochromic compounds in the vicinity of QDs for efficient Förster resonance energy transfer (FRET).^10,11^ However, silica shelling mostly relies on the particular reactivity of Si alkoxides, and the synthetic approach is hardly translatable to other metal oxides.^13^ More generally, controlling the thickness of these coatings is challenging, and strategies to obtain subnanometric thicknesses are limited.^14,15^

As an alternative to previous methods to grow QD-based hybrid materials, our group has developed a solution-based approach, termed colloidal atomic layer deposition (c-ALD). C-ALD enables the growth of hybrid ligand/metal-oxide shells of tunable thickness around different nanocrystalline cores.^16−20^ Hybrid materials incorporating QDs and photoactive ligands, which are important for applications in photocatalysis and photon upconversion, were recently synthesized via c-ALD enabling the preparation of funnel structures that would be obtained with difficulty via conventional ligand exchange methods.^20^ The c-ALD-grown shells also enhance the colloidal stability of nanocrystal inks by locking their native ligands on the surface.^18,19^ Furthermore, the deposition of these shells has enabled the study of fundamental processes in perovskite nanocrystals such as anion exchange and distance-dependent energy transfer.^16,17^

Most studies on QDs have thus far focused on Cd- and Pb-containing QDs.^21^ However, the QD field is experiencing a shift toward nontoxic materials to reach consumer markets.^22^ As such, our aim is to extend the library of QD-based hybrid materials to Restriction of Hazardous Substances directive (RoHS)-compliant compositions.

Indium phosphide (InP) QDs have arisen as the leading alternative to Cd-containing QDs in the visible region as they offer promising intrinsic optical properties (e.g., comparable extinction coefficient to Cd-based QDs, large Bohr exciton radius and wide emission range (1.9–2.5 eV)).^23,24^ Several approaches to the synthesis of InP QDs are reported in the literature.^24−27^ Tris(trimethylsilyl)phosphine [(TMS)_3_P] is one of the most used phosphorus precursors, in combination with fatty acid salts of indium, to obtain QD ensembles featuring narrow emission lines of 40–60 nm full width at half-maximum (fwhm) and near-unity photoluminescence quantum yields (PLQY). In recent years, efforts have focused on developing InP QD syntheses that avoid the use of costly and pyrophoric [(TMS)_3_]P.^26^ These efforts have resulted in synthetic approaches that rely on the redox chemistry of aminophosphines, such as tris(dimethylamino)phosphine [(DMA)_3_P] and tris(diethylamino)phosphine [(DEA)_3_P].^28−31^ The reaction of these aminophosphines with indium halides successfully produces QD ensembles with state-of-the-art emission width lines (43–63 nm) and emission efficiencies.^28−31^

Although both synthetic approaches produce InP QDs, the different chemistries involved in the synthesis render QDs passivated by different moieties. The InP QDs obtained via the silylphosphine route are passivated with carboxylates; instead, the aminophosphine route has been shown to yield amine-halide co-passivated InP QDs.^32,33^ The understanding and control of the surface chemistry of QDs is crucial to manipulate the properties of the as-synthesized QD as well as for the growth of heterostructures where the interface plays a crucial role.^34,35^ This last point is particularly relevant when applying the c-ALD approach, as the nucleation of the shell is determined by the interaction of the organometallic precursors with the surface-bound functional groups.^19^

In this work, we investigate the growth by c-ALD of ZnO shells on InP QDs obtained by either the silylphosphine or aminophosphine route. ZnO was chosen as the shelling material because most of the previous efforts at surface passivation of InP QDs to enhance their <1% PLQY are based on Zn-chalcogenide shells or on surface treatment with Zn-based ligands.^31,36−39^ First, we highlight the different surface chemistries of as-synthesized InP QDs regarding ligand passivation and surface oxidation. We then probe the interaction of the shell precursor, dimethylzinc (DMZ), with the passivating ligands by means of ^1^H solution nuclear magnetic resonance (NMR) and photoluminescence spectroscopy. We discover that the addition of DMZ enhances the PLQY significantly only for the aminophosphine InP QDs and that this enhancement is maintained in the final InP@ZnO core@shell hybrids. Finally, we demonstrate that the shell confers improved stability under ambient conditions to the aminophosphine-based QDs, which otherwise undergo ligand displacement and precipitate.

## Results and Discussion

**Figure 1:**
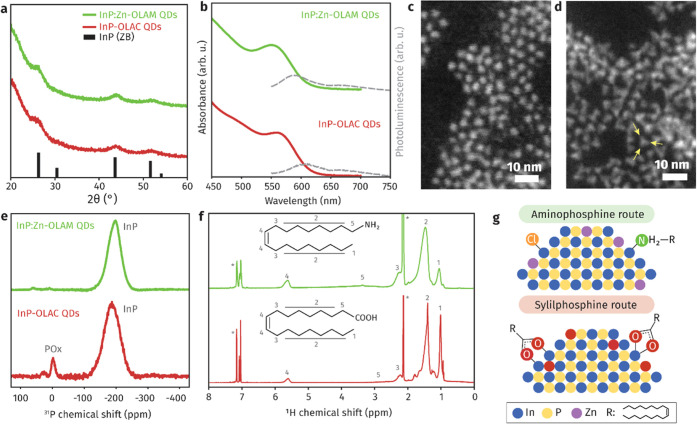
Characterization of the as-synthesized InP QDs. (a) XRD pattern confirming the zincblende crystal structure. (b) UV–vis absorbance and PL spectrum (excitation wavelength at 420 nm). (c, d) Dark-field HAADF-STEM image of (c) spherical InP-OLAC QDs and (d) tetrahedral InP:Zn-OLAM QDs; yellow arrows point to the vertices of one nanocrystal as a guide to the eye. (e) ^31^P{^1^H} MAS NMR spectra of the as-synthesized QDs. (f) ^1^H NMR spectrum in toluene-d_8_ showing broad resonances corresponding to bound oleate (InP-OLAC) and oleylamine (InP:Zn-OLAM); residual solvent resonances are labeled with (*). Red lines correspond to InP-OLAC QDs, and green lines correspond to InP:Zn-OLAM QDs. (g) Schematic illustrations of the surface ligand and composition of InP QDs obtained with [(TMS)_3_P] and [(DEA)_3_P].

Published protocols for the silylphosphine and aminophosphine routes were employed to synthesize zincblende InP QDs (Figure 1a).^29,40^ In the former, indium acetate and [(TMS)_3_P] were reacted in ODE and OLAC to obtain the samples that we refer to as InP-OLAC QDs.^40^ In the latter, InCl_3_ and [(DEA)_3_P] were mixed in OLAM in the presence of ZnCl_2_ to generate InP:Zn-OLAM QDs which possess a Zn-doped surface (Table S1), in agreement with the literature.^29,30^

The band-edge position in the ultraviolet–visible (UV–vis) absorption spectra for InP-OLAC and InP:Zn-OLAM QDs (Figure 1b) is at 555 and 563 nm, respectively, which corresponds to nanocrystals with a diameter of around 3.5 nm, consistently with the size obtained by electron microscopy (Figures 1c,d, S1, and S2, Table S2). We note that InP:Zn-OLAM QDs appear with a projected triangular shape, while InP-OLAC QDs are more spherical, in agreement with previous observations.^36,41^

The ^31^P{^1^H} solid-state NMR spectrum of both QDs shows a major contribution at −197 ppm, which corresponds to nanoparticulate InP (Figure 1e).^34,42−44^ The minor resonances in the interval 60–0 ppm are related to phosphorus atoms located at or near the surface (Figure S3) and have been previously assigned to different species of phosphorus oxides.^34,43,44^ The integrated fraction of ^31^P signal corresponding to phosphorus oxide represents 4% of the total ^31^P signal for InP-OLAC while it is marginal for InP:Zn-OLAM.

Quantitative ^1^H NMR spectroscopy (Figure 1f) evidences that oleate and oleylamine ligands passivate the surface of the InP-OLAC QDs and InP:Zn-OLAM QDs with a respective ligand density of 4.5 oleate/nm^2^ and ∼2 oleylamine/nm^2^ (see the Supporting Information (SI) for details). Chloride is also present on the InP:Zn-OLAM QDs (Figure S4 and Table S3) resulting in amine-halide co-passivated InP QDs, as previously reported.^30,33^Figure 1g provides a schematic of the surface chemistry and shows the differences between the QDs obtained with the two approaches.

**Figure 2:**
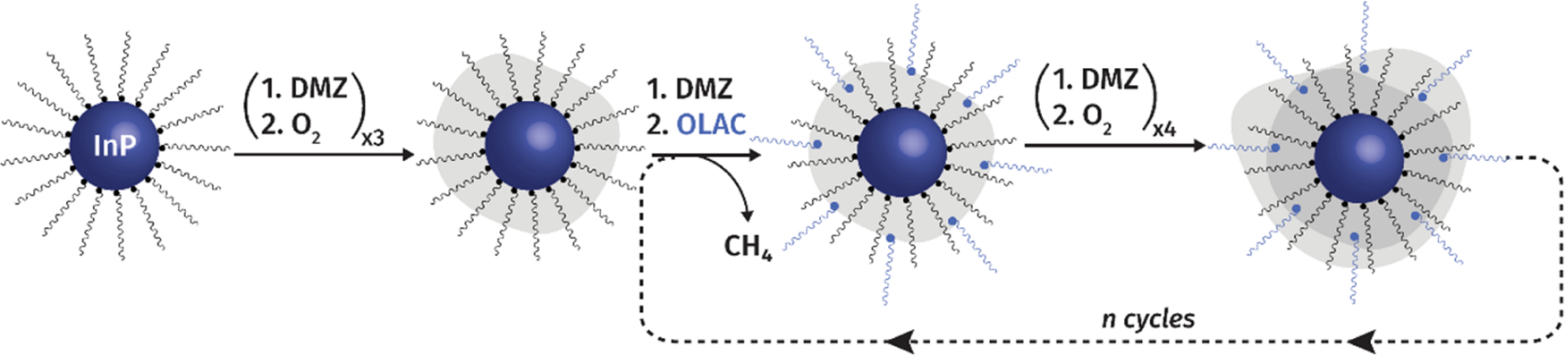
Schematic illustration of the c-ALD process. Injections of DMZ and oxygen alternate during the process. Periodic additions of OLAC, instead of oxygen, are performed to preserve the colloidal stability.

Having gained information on the surface of the as-synthesized InP QDs, we evaluated the eventual changes in the identified moieties during the nucleation and the growth of the ZnO shell via the c-ALD approach, schematically depicted in Figure 2. The process begins with the addition of an organometallic complex, which is dimethylzinc (DMZ) in the present case, and continues by alternating additions of oxygen and of DMZ. Periodically, the oxygen step is substituted by the injection of OLAC, in order to preserve the colloidal stability of the hybrids (see the SI for details).

**Figure 3:**
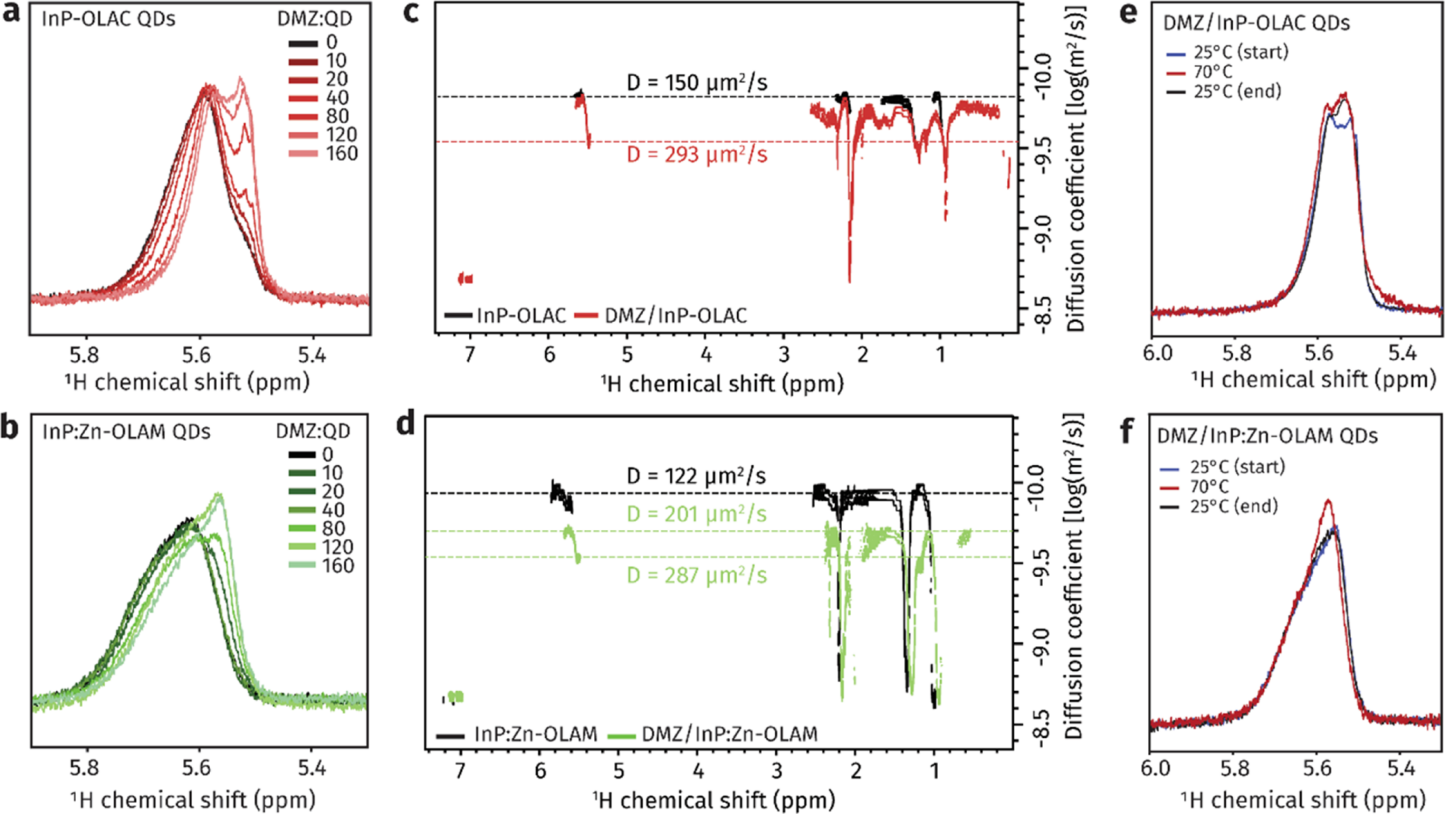
Study of the interaction of DMZ with surface ligands on the InP QDs (i.e., shell nucleation). (a, b) ^1^H NMR of the alkene resonance of (a) the native oleate ligands and (b) the native oleylamine ligands throughout the addition of increasing DMZ equivalents. (c, d) 2D-DOSY spectra of (c) InP-OLAC QDs (black) and DMZ:(InP-OLAC) = 120 (red) and (d) InP:Zn-OLAM QDs (black) and DMZ:(InP:Zn-OLAM) = 120 (green). (e, f) ^1^H VT NMR of the alkene resonance after the addition of DMZ at room temperature (blue), at 70 °C (red), and at room temperature again upon cooling (black) for (e) DMZ:(InP-OLAC) = 120 and (f) DMZ:(InP:Zn-OLAM) =120.

Figure 3 summarizes the results obtained via NMR spectroscopy to gain insight into the interaction of DMZ with the surface ligands. Figure 3a,b shows the ^1^H NMR spectrum of the alkene resonance of the oleate (Figures 3a and S5) and of the oleylamine (Figures 3b and S5), which are on the surface of the In-OLAC and InP:Zn-OLAM, respectively. We used this region to monitor the reactivity of the ligands with DMZ upon titration to the QD suspension. A new resonance upfield to that of the bound alkene proton emerges for the In-OLAC since the start of the titration and increases in intensity with increasing DMZ equivalents (Figures 3a and S6), which indicates that the oleate ligands readily react with DMZ. Concomitantly, the diffusion ordered NMR spectroscopy (DOSY) of InP-OLAC shows that the diffusion coefficient (*D*) transitions from a single population with *D* = 150 μm^2^/s to two distinct diffusion coefficients, one at the original *D* = 150 μm^2^/s and the other at 293 μm^2^/s for DMZ/InP-OLAC, where 120 equiv of DMZ were added to the InP-OLAC QDs (Figure 3c). This result indicates that the interaction of one fraction of the oleates with DMZ generates a new chemical species with a larger diffusion coefficient, which is thus either in a dynamic equilibrium with the surface of the InP QDs or completely free in solution. We used variable temperature (VT) NMR to gain further insight into the binding state of the new species generated from the reaction of OLAC and DMZ. Upon heating the sample from 25 to 70 °C, the resonance at 5.5 ppm increases in intensity, and this feature is maintained upon cooling (Figures 3e and S7). This result indicates that the oleate and DMZ endothermically react to form a ligand–precursor complex, as suggested also by the ^1^H NMR of DMZ and OLAC mixtures in solution (Figure S8), similarly to our previous study.^19^ A fraction of this complex binds to the QD surface, while the remaining fraction is free in solution.

In striking difference, changes in the bound oleylamine resonance of the InP:Zn-OLAM are observed only after 40 equiv of DMZ are added (Figures 3b and S5), which indicates a lower reactivity of the OLAM toward DMZ. Instead of an upfield resonance distinctive from the original ligands, the OLAM alkene resonance progressively shifts upfield as a whole and changes its shape. The DOSY spectra show two distinct diffusion coefficients, one at 201 μm^2^/s and the other at 287 μm^2^/s after the addition of 120 equiv of DMZ (Figure 3d). Similarly to the In-OLAC, the latter indicates the presence of one new, more rapidly diffusing, species. However, the original diffusion coefficient (*D* = 122 μm^2^/s) is not preserved in this case, pointing to the participation of the totality of the ligand shell in the interaction with the DMZ, instead of only one fraction as observed for the In-OLAC. The VT NMR shows a significant sharpening of the line shape at 70 °C, but the original features are completely recovered upon cooling (Figures 3f and S9). This result indicates that the new species forming from DMZ and OLAM are in a dynamic exchange with the oleylamine passivating the QDs. The ^1^H NMR spectra of DMZ and OLAM mixtures suggest the formation of coordination complexes (Figure S10), consistent with previous studies.^45,46^ We therefore conclude that the addition of DMZ forms Zn(Me)_2_(oleylamine), which is dynamically bound to the QDs.

Thus, the study of the first half-cycle of the c-ALD process let us conclude that DMZ reacts with both oleate and oleylamine, albeit the oleylamine has a lower reactivity than the oleate, and the complex formed in each case has a different chemical interaction with the QD surface.

**Figure 4:**
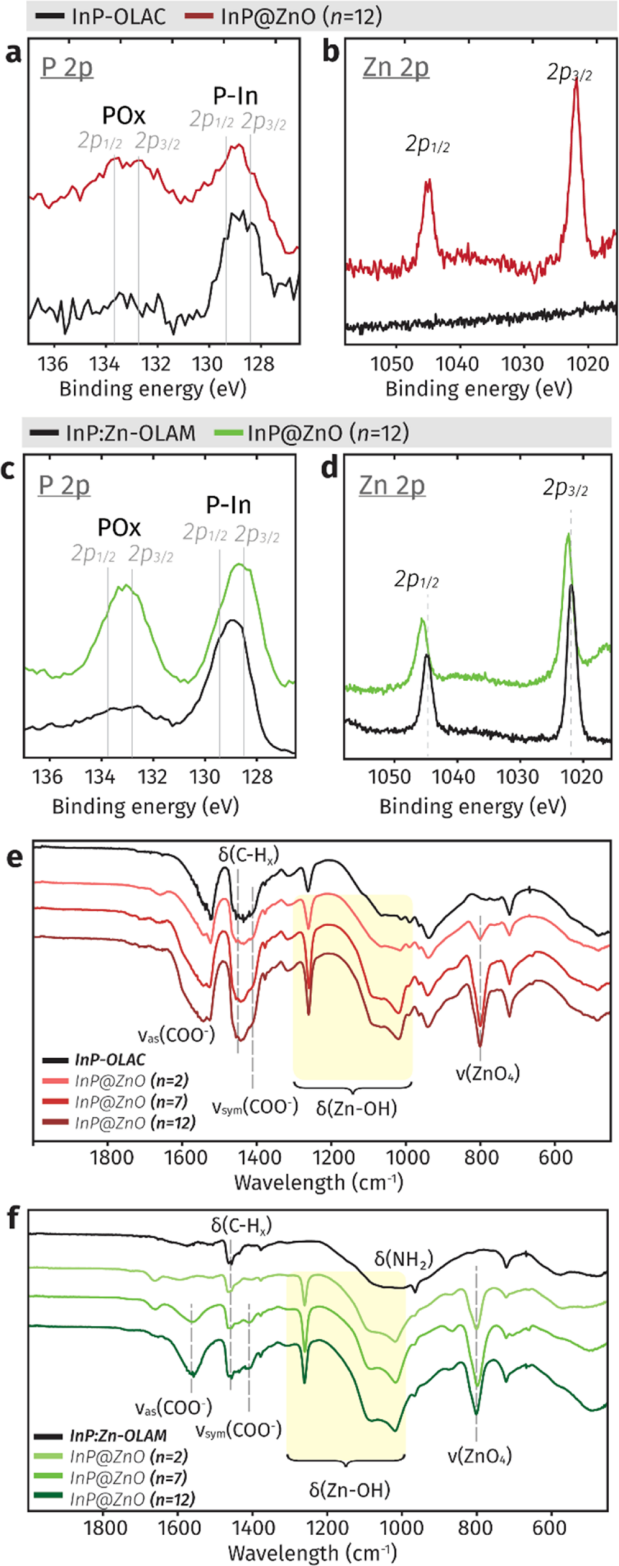
Surface characterization of the InP@ZnO hybrid QDs synthesized via c-ALD (i.e., shell growth). (a–d) XPS spectra of (a, b) InP-OLAC and (c, d) InP:Zn-OLAM with their respective core@shell systems for regions corresponding to (a, c) P 2p and (b, d) Zn 2p. (e, f) FTIR spectra of (e) InP-OLAC QDs and (f) InP:Zn-OLAM with their respective core@shell hybrid QDs.

Continuing with the shell growth, X-ray photoelectron spectroscopy (XPS) analysis (Figure 4a–d) indicates a significant increase of the POx in both QDs, which hints at the formation of InPOx as an interfacial layer between InP and the ZnO shell (Figure 4a,c). The appearance of the Zn 2p peaks in InP-OLAC QDs (Figure 4b) is consistent with the shell growth. The Zn 2p region of the as-synthesized InP:Zn-OLAM (Figure 4d) shows the presence of Zn as a result of the synthetic route. After c-ALD, the peaks shift toward higher binding energy and become broader, as Zn–O contributions are expected at higher binding energy than Zn–P.^47^ The XPS results are in agreement with the XANES and EXAFS data collected at the In and Zn K-edge (Figures S11–S13, Tables S4 and S5, see the SI for details). The Zn edge in both QDs clearly indicates the formation of Zn–O bonds following shell growth. Particularly interesting in both systems is the emergence of a significant contribution of octahedrally coordinated indium upon shell growth, which is consistent with the presence of an interfacial layer of InPOx. This result falls in line with previous reports indicating that InP can accommodate oxygen beyond preservation of the overall QD charge neutrality that may lead to a suprastoichiometric surface composition, without implying the full oxidation of the nanocrystal core.^48^

The FTIR analysis (Figures 4e,f and S14–S15) further confirms the presence of ZnO after c-ALD in both systems, with new strong absorption bands appearing in the low-wavenumber region (750–1275 cm^–1^). The bands at 1258, 1087, and 1018 cm^–1^ correspond to bending of Zn–OH (highlighted in the yellow region), while we tentatively assign the resonance at 800 cm^–1^ to the stretching of ZnO_4_.^49−52^

In addition, FT-IR enables the detection of organic ligands before and after c-ALD. For as-synthesized InP-OLAC (Figure 4e), the presence of native OLAC is evident from the *v*_sym_ and *v*_as_ stretching modes of COO^–^. The wavenumber difference between *v*_sym_ and *v*_as_ (Δ) can be used to assess the binding mode of carboxylate ligands.^53,54^ We find that the native ligands are in a mixture of chelating and bridging coordination modes. After two cycles (*n* = 2), and before the incorporation of additional oleates, the asymmetric peak significantly broadens, pointing at the participation of the native ligands in the nucleation of the shell. The asymmetric peak continues to broaden with the number of cycles (Figure 4e) and incorporation of oleates, which indicates that the newly added oleates coordinate to the metal-oxide shell in a bridging mode (Figures S14 and S15).

For the as-synthesized InP:Zn-OLAM QDs (Figure 4f), the presence of OLAM is indicated by the band at 1460 cm^–1^ (δ(CH_2_)) and (δ(CH_3_)_as_), and a broad feature in the 1200–900 cm^–1^ region (δ(NH_2_)).^41^ The appearance of the symmetric (*ν*_sym_) and asymmetric (*ν*_as_) stretching modes of the COO^–^ moiety at 1460 and 1560 cm^–1^ at *n* = 7 and 12 indicates that most oleates added during c-ALD are in a bridging coordination mode.

Overall, the data discussed above indicate that the shell nucleation on InP-OLAC relies on the formation of ligand–precursor complexes, as observed before for oleate-capped CdSe QDs.^19^ Furthermore, the oxide surface species could also act as anchoring points for the shell nucleation, as previously found for other systems.^16^ The integration of the alkene resonance indicates that not all of the added DMZ interacts with the ligands (Figure S6), which is consistent with the hypothesis that a fraction reacts and nucleates directly on the oxide surface species.

On the contrary, the lack of initial surface oxidation, the lower reactivity of OLAM with DMZ compared to OLAC, and the dynamic nature of the ligand–precursor complex formed upon addition of the DMZ suggest that the nucleation step is mediated by a different pathway for the InP:Zn-OLAM QDs.

While the chemistry involved in the nucleation step is different, a ZnO shell can be successfully grown by c-ALD on both InP QDs independent of the initial surface chemistry.

**Figure 5:**
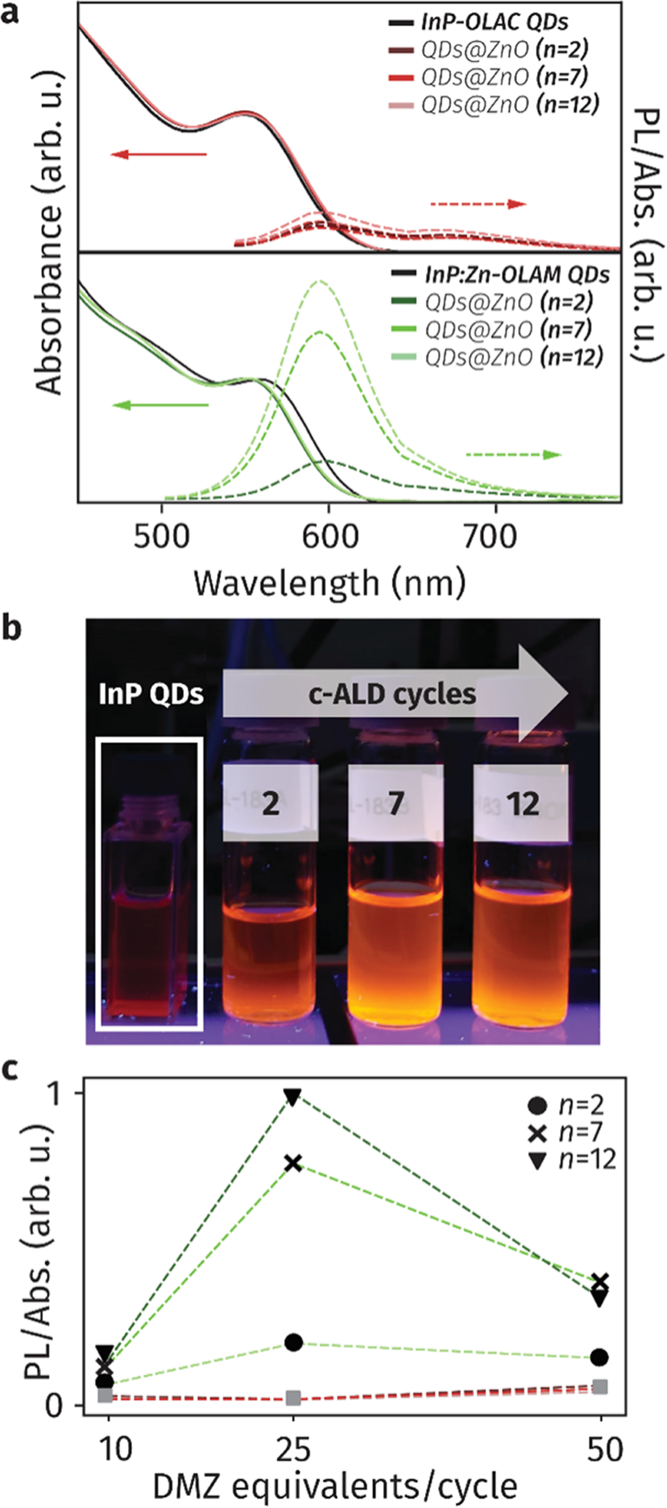
Optical properties of InP@ZnO hybrid QDs. (a) Normalized UV–vis spectra and corresponding PL emission (normalized to excitation wavelength, λ = 420 nm) of InP-OLAC QDs (top) and InP:Zn-OLAM (bottom) after 2, 7, and 12 cycles of c-ALD in which 25 equiv of DMZ per nanocrystal were added in each cycle. (b) Photograph of the InP:Zn-OLAM@ZnO QDs obtained with DMZ:QD = 25 for different ALD cycles under UV excitation. (c) PL intensity normalized to absorption as a function of the number of equivalents added per cycle and number of c-ALD cycles. For InP:Zn-OLAM QDs, full circles, crosses, and triangles (●,**×**,▼) indicate samples with 2, 7, and 12 c-ALD cycles, respectively. For InP-OLAC QDs, only gray squares (■) are depicted for simplicity, as different number of cycles did not result in enhanced PL emission. We note that residual trap emission remains despite increased PLQY in InP:Zn-OLAM QDs, which indicates various types of defects are involved, similarly to what was observed before for other surface treatments.^38^

We then investigated the impact of the c-ALD grown ZnO shell on the optical properties of the InP QDs (Figure 5). The band-edge transition and the photoluminescence emission of the InP-OLAC@ZnO hybrids do not vary significantly compared to that of the as-synthesized InP-OLAC, regardless of the number of cycles (Figure 5a, top). As for the InP:Zn-OLAM QDs, the band-edge transition undergoes minor blue shifts (10–20 nm, 40–80 meV) as the number of c-ALD cycles increases (Figure 5a, bottom), though these are not sufficient to indicate any significant change in QD size but rather indicate a change in the electronic properties. More interestingly, the PL emission of the InP:Zn-OLAM QDs increases upon ZnO shell growth, which is evident also in the photograph of the samples under UV excitation (Figure 5b). With the aim of further understanding the source of this promising enhancement, we varied the DMZ:QDs ratio added in each cycle (Figure S16). While we observed no difference for the InP-OLAC QDs, an optimal number of DMZ molar equivalents (<40) which maximizes the PLQY exists for the InP:Zn-OLAM QDs (Figure 5c).

In order to rationalize the different changes in PL emission, we recall the initial surface characterization. The as-synthesized InP-OLAC QDs are initially surface oxidized and passivated by oxygen-containing ligands. Both moieties will act as anchoring points for the nucleation of metal-oxide shells, in line with previous observations from our group.^16,19^ On the other hand, InP:Zn-OLAM QDs present a structure of purely In–P in tetrahedral coordination, with no significant surface oxidation. Recently, Ubbink and co-workers reported that a Z-type ligand treatment on InP QDs was less effective in enhancing the PLQY of polyphosphate-passivated QDs versus those polyphosphate-free.^44^ We suggest that the addition of DMZ (which can be considered a Z-type ligand) only benefits those QDs that do not have intrinsic surface oxidation, and therefore the PLQY increases only for the case of InP:Zn-OLAM. Regarding the optimal number of DMZ molar equivalents (<40) that maximizes the PLQY, the ^1^H NMR titration data indicated the formation of the labile Zn-OLAM complex around the same number of DMZ equivalents. Thus, the DMZ must be effectively passivating trap sites on the surface of the InP:Zn-OLAM QDs until the point when it starts perturbing the original ligand shell.

Going a step further to speculate on the type of defects passivated by the DMZ on the InP:Zn-OLAM, we focus on one additional difference between the two systems, which is the shape and thus the facets exposed. While silylphosphine routes generally yield spherical nanocrystals, aminophosphines form tetrahedral InP QDs.^24,30^ The tetrahedral nanocrystals are In(111) terminated for “large” edge size (≈10 nm), but smaller sizes (<5 nm) present a mixture of mostly In(111) and (110) facets, with truncations on the In(100) and P(−1–1–1) facets.^30,41,55^ The difference in shape of the InP-OLAC and InP:Zn-OLAM are observed in the STEM images (Figure 1c,d) and translate into the ICP-OES results (Table S1), which evidence a higher phosphorus-to-metal ratio for the InP:Zn-OLAM. We propose that the presence of P(−1–1–1) facets alongside the oxide-free nature of the InP:Zn-OLAM QDs facilitates a Z-type passivation of undercoordinated surface P atoms by DMZ and a concomitant increase of the PLQY. This increase in PLQY is evident as long as the equivalents of DMZ are low enough to avoid disrupting the ligand shell, which thus sets the upper limit to the number of DMZ equivalents to add during the first ALD cycle for optimal properties of the final InP@ZnO hybrid QDs. Based on the optical data, we thus propose that the undercoordinated P atoms offer an anchoring point for the nucleation of the shell on the InP:Zn-OLAM QDs.

On the contrary, the InP-OLAC QDs with their initially oxidized surfaces and with larger In:P ratio by ICP are less likely to contain undercoordinated surface P atoms that may be passivated by Zn complexes.

**Figure 6:**
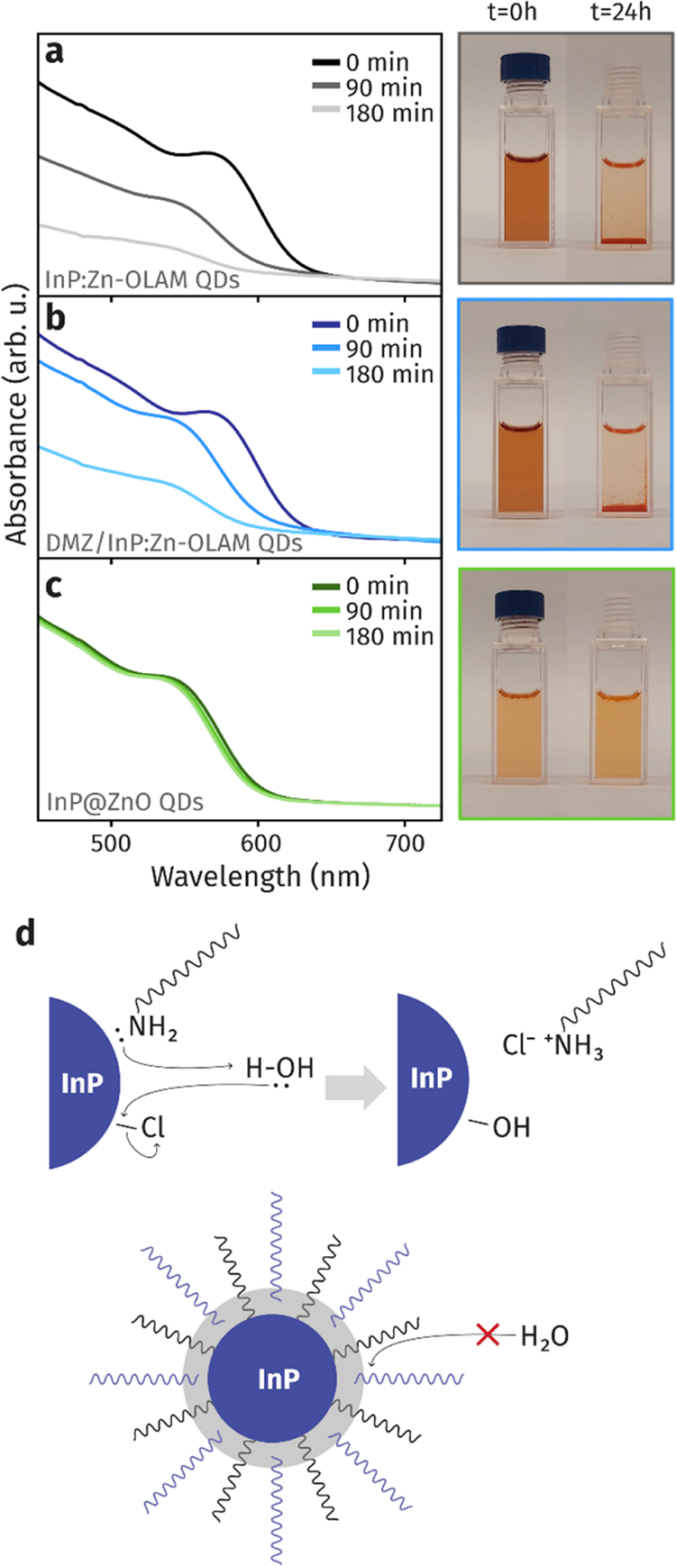
Stability under ambient conditions. (a–c) UV–vis absorption spectra of (a) InP:Zn-OLAM QDs, (b) InP:Zn-OLAM QDs treated with 25 equiv of DMZ, and (c) InP:Zn-OLAM@ZnO QDs (*n* = 12 cycles, DMZ:QDs = 25) over a period of 3 h. On the right, photographs of the suspensions and precipitates measured over a period of 24 h. (d) Schematics of the proposed effect of ambient water on the surface of InP:Zn-OLAM QDs, which causes their colloidal instability,^55^ and of the impact of the ZnO, which prevents water to reach the native OLAM, thus enhancing the stability of the QDs.

Alongside the optical properties, we investigated the colloidal stability of the newly synthesized core@shell materials when exposed to ambient conditions. While InP-OLAC QDs are generally stable, InP:Zn-OLAM QDs lose their colloidal stability when exposed to ambient conditions.^55^ Indeed, we found that the InP:Zn-OLAM QDs fully precipitate after only 90 min of exposure, as tracked by the decrease of absorbance of the suspension over time (Figure 6a). A similar outcome is observed for InP QDs surface treated with 25 equiv of DMZ (Figure 6b). We explain the slightly prolonged stability of the DMZ-treated InP:Zn-OLAM QDs compared to the as-synthesized sample with the in situ formation of a ZnO matrix due to the reaction of DMZ with ambient O_2_ and H_2_O. Thus, even if a surface treatment with DMZ alone might be sufficient to enhance the PL of the InP:Zn-OLAM QDs, the obtained QDs would not be stable when exposed to ambient conditions. On the contrary, the InP:Zn-OLAM@ZnO QDs exhibit increased stability on the time scale of these measurements (24 h) (Figure 6c). Furthermore, this sample shows bench stability in ambient conditions for up to several months. We found that samples prepared with *n* = 2 c-ALD cycles were not stable under ambient conditions and that at least *n* = 7 cycles were needed to obtain a suspension of InP:Zn-OLAM@ZnO, which was stable for extended periods.

The mechanism currently proposed to explain the instability of In(As,P) QDs is that water protonates the surface-bound OLAM ligands and, as such, induces the formation of surface hydroxyl groups, the desorption of an oleylammonium chloride ion pair and the eventual oxidation and loss of colloidal stability of the QDs (Figure 6d).^55^ We suggest that the stability observed in samples with *n* > 7 is the result of a synergistic effect of possessing a thicker ZnO shell, which blocks water from protonating the ligands, as well as the incorporation of oleate ligands in the metal oxide (Figure 6d).

## Conclusions

In conclusion, we developed the synthesis of novel hybrid materials including InP QDs and an organic/inorganic ZnO shell via c-ALD. We demonstrated the importance of the surface chemistry of the InP QDs in the shell nucleation mechanism, as well as for the optical properties of the final materials. In particular, we compared InP QDs obtained by the conventional silylphosphine route (InP-OLAC) and those obtained by the more recently developed aminophosphine route (InP:Zn-OLAM) and discovered that only the InP:Zn-OLAM@ZnO hybrids possess an enhanced PLQY compared to the as-synthesized material. We proposed the distinct interaction of the c-ALD precursor DMZ with the OLAM ligands and the ability of the DMZ to passivate defects, which are unique to the InP:Zn-OLAM QDs, as the main factors behind the PL enhancement. In the future, different c-ALD precursors, including metal–alkyls and other precursors generally used in gas-phase ALD, such as metal-amines, may help to find the right balance between the chemistries needed to enable shell formation, passivate the InP surface, and enhance further the PL emission. Finally, we demonstrate the importance of the ZnO shell in enhancing the colloidal stability of the InP:Zn-OLAM under ambient conditions.

Overall, this work proposes c-ALD as a methodology for the synthesis of alternative InP core@shell QDs with improved stability for application in optoelectronic devices and bioimaging.

## Experimental Methods

### Chemicals

Indium acetate (In(CH_3_COO)_3_, 99.99% trace-metal basis), indium chloride (InCl_3_, 99.99% trace-metal basis), tris(trimethylsilyl)phosphine ([(TMS)_3_]P, 95%), tris(diethylamino)phosphine ([(DEA)_3_]P, 97%), oleic acid (OLAC, 90% technical grade), oleylamine (OLAM, 70% technical grade), ethanol (EtOH, anhydrous, 95%), octane (anhydrous ≥99%), octadecene (ODE, 90% technical grade), toluene-*d*_8_ (Tol-*d*_8_, 99.5 atom % D), and dimethylzinc (DMZ, 1.2 M in toluene) were purchased from Sigma-Aldrich. Hexane (anhydrous, >96%) was purchased from TCI. Zinc chloride (ZnCl_2_, >98%) was purchased from Alfa Aesar.

#### Colloidal Atomic Layer Deposition

The DMZ stock solution (1.2 M in toluene) was purchased from Sigma-Aldrich and used without further purification. The DMZ solution employed for c-ALD was prepared by diluting the stock DMZ with anhydrous octane to yield a 3.2 mM solution. This solution was then transferred to a gastight syringe (S.G.E. Gas Tight Luer Lock Syringe 5 mL) with a stainless steel 304 needle from Sigma-Aldrich inside a nitrogen-filled glovebox.

A 10 mL suspension of 5 μM QDs in anhydrous octane was prepared from the stock QDs suspension inside a nitrogen-filled glovebox. This suspension was then transferred to a three-neck flask that had been previously purged with nitrogen. The nitrogen was supplied through a three-way valve to ensure the constant renewal of nitrogen and replacement of the introduced O_2_ and generated gas byproducts (methane). The suspension was constantly stirred at room temperature for the duration of the experiment.

The first half-cycle of one c-ALD cycle involves the injection of 440 μL of the DMZ solution (for 28 equiv of DMZ:QDs) at a rate of 1 mL/h using a syringe pump (Chemyx Fusion 200 two-channel injection pump). The DMZ injection was followed by 5 min of waiting time and then the bubbling of O_2_, by means of a mass flow controller at a rate of 1.5 mL/min for 1 min. To prevent the loss of colloidal stability, at cycles *n* = 4 and *n* = 9, OLAC ligands were introduced in place of O_2_ bubbling, using a purged needle, and directly injected into the QDs suspension. A stoichiometric amount of 1:1 with respect to DMZ was introduced from a solution of OLAC in anhydrous octane.

We can express the completion of n c-ALD cycles as *n*·([DMZ/O_2_]), and the cycles where OLAC is added as ([DMZ/OLAC]). Therefore, we can summarize the processes to obtain the samples discussed in the main text as

(a) *n* = 2 → 2([DMZ/O_2_])
(b) *n* = 7 → 3[DMZ/O_2_] + [DMZ/OLAC] + 3[DMZ/O_2_]
(c) *n* = 12 → 3[DMZ/O_2_] + [DMZ/OLAC] + 4[DMZ/O_2_] + [DMZ/OLAC] + 3[DMZ/O_2_]

After the desired number of cycles, the solution was either dried under a constant flow of nitrogen or washed by precipitation and centrifugation with an antisolvent (EtOH). The QDs were then redispersed in the desired anhydrous solvent, such as deuterated toluene or octane, and stored in a nitrogen-filled glovebox.

The gastight syringe was then washed by flowing 15 mL of anhydrous hexane to avoid alumina formation and clogging of the syringe. The syringe and needle were then placed in a 60 °C oven. If oxide deposits were observed, the syringe was charged with a 1 M HCl solution and left for 1 h. The syringe was then thoroughly rinsed with water and stored in the oven.

#### InP-OLAC QDs

InP-OLAC QDs were synthesized by adapting published methods.^40^ Indium acetate (1.17 g, 4 mmol) and oleic acid (4.10 g, 14.5 mmol) were mixed in 1-octadecene (4 g) and degassed at 100 °C overnight. Upon heating the reaction flask to 315 °C under an inert atmosphere (N_2_(g)), P(SiMe_3_)_3_ (2 mmol) suspended in 1-octadecene (4 g) was injected. After the phosphorus injection, the reaction proceeded at 300 °C for 20 min. At the end of the reaction, the flask was cooled down by means of an ice bath. The InP nanocrystals were then purified by repeated precipitation and redissolution cycles using hexane and ethanol as the solvent and antisolvent, respectively.

#### InP:Zn-OLAM QDs

InP:Zn-OLAM QDs were synthesized following published methods.^29^ Indium chloride (100 mg, 0.45 mmol) and zinc chloride (300 mg, 2.2 mmol) were mixed in technical grade oleylamine (5 mL, 15 mmol). The reaction mixture was degassed at 120 °C for 1 h and then heated to 180 °C under inert atmosphere (N_2_(g)). Upon reaching 180 °C, a mixture of tris(diethylamino)phosphine [(DEA)_3_P] (0.45 mL, 1.6 mmol) in oleylamine (2 mL) was quickly injected into the above mixture. After the phosphorus precursor injection, the reaction proceeded at 180 °C for 25 min. At the end of the reaction, the flask was cooled down by means of an ice bath. The InP nanocrystals were then purified by repeated precipitation and redissolution cycles using hexane and ethanol as the solvent and antisolvent, respectively.

For this synthetic approach, the use of ZnCl_2_ was demonstrated to produce smaller QDs and to reduce the size dispersion of the QDs ensembles, which dictated our choice of the procedure.^33,41^

## Data Availability

The data underlying this study are openly available in Zenodo at 10.5281/zenodo.8377955.

## Supporting Information Available

Additional experimental details; FTIR, XPS, NMR, TEM, UV–vis, and XAS; and further discussions (PDF)
