# Supplementary material for: Surface Chemistry Dictates the Enhancement of Luminescence and Stability of InP QDs upon c-ALD ZnO Hybrid Shell Growth
Source: JACS Au. 2023 Nov 1;3(11):3066–75. doi: 10.1021/jacsau.3c00457 (PMC10685429; doi:10.1021/jacsau.3c00457)
Supplement: Supplementary file 1 — au3c00457_si_001.pdf [file au3c00457_si_001.pdf]

Supporting information

Surface chemistry dictates the enhancement of luminescence and stability of InP QDs upon c-ALD ZnO hybrid shell growth

Ona Segura Lecina[1], Mark A. Newton[1], Philippe B. Green[1], Petru P. Albertini[1], Jari Lemmans[1], Kenneth P. Marshall[2], Dragos Stoian[2], Anna Loiudice[1], Raffaella Buonsanti[*,1]

[1]Laboratory of Nanochemistry for Energy (LNCE), Institute of Chemical Sciences and Engineering (ISIC), École Polytechnique Fédérale de Lausanne, CH-1950 Sion, Switzerland.

[2]The Swiss-Norwegian Beamlines, European Synchrotron Radiation Facility (ESRF), 38000 Grenoble, France.

* Corresponding author: raffaella.buonsanti@epfl.ch

Table of contents

Experimental Section: Characterization 3

References 30

Figure S1: Absorbance fitting of as-synthesized InP QDs 9

Figure S2: HAADF-STEM of as-synthesized InP QDs 10

Figure S3: ^{31}P CP/MAS of as-synthesized InP QDs 11

Figure S4: XPS 12

Figure S5: ^{1}H NMR full spectra of InP-OLAC and InP:Zn-OLAM DMZ titration 15

Figure S6: ^{1}H NMR alkene signal fitting of InP-OLAC and InP:Zn-OLAM DMZ titration 16

Figure S7: VT ^{1}H NMR of as-synthesized InP-OLAC and DMZ/InP-OLAC QDs 17

Figure S8: ^{1}H NMR of DMZ–OLAC reaction in solution 18

Figure S9: VT ^{1}H NMR of as-synthesized InP:Zn-OLAM and DMZ/InP:Zn-OLAM QDs 19

Figure S10: ^{1}H NMR of DMZ–OLAM reaction in solution 20

Figure S11: EXAFS 21

Figure S12: XANES and k^3 weighted fittings for EXAFS (InP:Zn-OLAM QDs) 23

Figure S13: XANES and k^3 weighted fittings for EXAFS (InP-OLAC QDs) 25

Figure S14: FTIR of OLAC, InP-OLAC QDs and corresponding InP@ZnO QDs 27

Figure S15: FTIR of OLAM, InP:Zn-OLAM QDs and corresponding InP@ZnO QDs 28

Figure S16: UV-vis absorption and PL emission for different DMZ:QD equivalents and number of cycles 29

Table S1: ICP-OES 8

Table S2: fitting values for UV-vis absorption of as-synthesized InP QDs 9

Table S3: fitting values for XPS 13

Table S4: EXAFS fitting parameters for InP-OLAC 24

Table S5: EXAFS fitting parameters for InP:Zn-OLAM 26

Experimental section.

Characterization. UV–vis absorption measurements were performed in a Perkin Elmer Lambda 950 Spectrophotometer equipped with deuterium and tungsten halide light sources and a photomultiplier tube with Peltier-controlled PbS detector. Colloidal suspensions of QDs in octane were measured in quartz cuvettes.

XPS measurements were recorded using a Kratos Analytical instrument, using the monochromatic Kα X-ray line of an Al anode. The pass energy was set to 20 eV with a step size of 0.1 eV. The samples were prepared by drop-casting nanocrystal films onto clean Si substrates. The samples were electrically insulated from the sample holder and charges were compensated. Curve fitting was performed using the CasaXPS software. Spectra were referenced at 284.8 eV using the C-C bond of the C1s orbital.

FTIR measurements were carried out on an attenuated total reflectance (ATR) PerkinElmer Two spectrometer. Samples dispersed in hexane were directly deposited by drop-casting on the ATR plate after measuring the background with air. The resolution used to acquire the spectra was 4 cm^{-1}.

HAADF-STEM imaging and energy dispersive X-ray analysis (EDX) were performed on a FEI Tecnai Osiris TEM in scanning mode at an accelerating voltage of 200 kV. This microscope is equipped with a high brightness X-FEG gun, silicon drift Super-X EDX detector and a Bruker Esprit acquisition software. Samples were drop-casted on a gold TEM grid (Ted Pella, Inc.) prior to imaging.

Solution NMR measurements were recorded on a Bruker Avance III HD 400 MHz 9.4 T spectrometer equipped with a BBFO liquid probe. One dimensional (1D) ^{1}H and 2D DOSY spectra were acquired using a standard pulse sequence from the Bruker library.

^{31}P solid-state NMR spectra were recorded on a 400 MHz Bruker spectrometer (9.4 T) equipped with an Avance III HD console and a 3.2 mm three-channel low temperature MAS probe.

Characterization details

<u>*Size and concentration determination by UV–vis absorption*</u>

The size of the QDs was determined using the following equation (1) proposed by Cho and co-workers:[5]

$$E_g\ (\mathrm{eV}) = 1.401 + \frac{3.493}{d^{1.172}} \qquad (1)$$

where the value for E_g was chosen from the position of the first excitonic peak expressed in eV, which was obtained from fitting a gaussian function into the absorbance spectra.
The molar attenuation coefficient (ε) of the QDs solution was calculated from equation (2), representing the empirical trend reported by Xie and co-workers:[6]

$$\varepsilon\ (M^{-1}cm^{-1}) = 17447.5 \cdot \lambda - 8515144.5 \qquad (2)$$

where λ is the position of the first excitonic peak expressed in nm.

$$[InP\ QDs](\mathrm{M}) = \frac{Abs}{l(\mathrm{cm})\varepsilon(\mathrm{M^{-1}cm^{-1}})} \qquad (3)$$

^{1}H nuclear magnetic resonance (NMR)

The quantitative 1D ^{1}H spectra were recorded with a 90 s relaxation delay to allow full relaxation of the internal standard (*ref*) (dibromomethane, $T_1 \approx 19$ s). The concentration of the species of interest (C_x) was calculated using equation (4), where C_{ref} corresponds to the molar concentration of the internal standard, *I* to the integral area and *N* to the number of nuclei producing the signal. Integration values were obtained by multi-line fitting in MestreNova.

$$C_\mathrm{x} = \frac{I_\mathrm{x}}{I_\mathrm{ref}} \cdot \frac{N_\mathrm{ref}}{N_\mathrm{x}} \cdot C_\mathrm{ref} \qquad (4)$$

For InP-OLAC QDs, the ligand density on the surface was calculated by assuming that the QD have a spherical shape with a diameter (*d*), as measured by TEM, thus with a surface area corresponding to $A = 4\pi(d/2)^2$, and that the molar concentration of QDs is calculated with equation (2). This rendered a surface ligand density of 4.5 oleate/nm^2.

For InP:Zn-OLAM QDs, with their truncated tetrahedral shape, it is harder to access the surface area per nanocrystal. If the two extreme cases are taken, 1) assuming it is a spherical particle and 2) assuming it is a perfect tetrahedron, the obtained ligand densities are 2.5 and 1.4 oleylamine/nm^2 respectively. We consider, therefore, an intermediate ligand density of ~2 oleylamine/nm^2.

Diffusion Ordered Spectroscopy (DOSY)

DOSY experiments were performed with a pulse field gradient spin-echo PFGSE decay (pulse sequence ledbpgp2s from the Bruker library). The diffusion parameters, consisting of the gradient pulse length δ/2 and the diffusion delay Δ, as well as the gradient strength, were optimized such that a signal decay of roughly 90 % was obtained at the highest gradient strength used. The gradient strength was varied using a smoothed squared gradient shape. The diffusion coefficient (*D*) extracted from these measurements enables the calculation of the hydrodynamic diameter (d_{H}) of the nanoparticles employing the Stokes–Einstein equation:

$$D = \frac{K_B T}{6\pi\eta d_{\mathrm{H}}} \qquad (5)$$

K_B is the Boltzmann constant, T corresponds to 298 K and η is the viscosity of the solvent. For Toluene-d_8, $\eta = 0.56\cdot10^{-3}$ Pa·s.

^{31}P solid-state NMR

Samples were packed into 3.2 mm zirconia rotors under ambient conditions and spun up to 20 kHz spinning speed using nitrogen gas. ^{31}P chemical shifts were referenced relative to 85% H_3PO_4 using the secondary reference $NH_4H_2PO_4$ at 1.33 ppm.[7]
In the one-pulse experiments, ^{31}P nuclei were excited with π/2 pulses of 3.8 μs with a recycle delay of 150 s, which is equivalent to 5*T_1.

^{31}P cross-polarisation (CP) MAS spectra were obtained by transferring polarization transfer from ^{1}H to ^{31}P followed by a Hahn echo on the ^{31}P. The spectra were recorded using variable amplitude during contact times of 0.5, 1.5 or 3 ms.[8] Echo delays were set to one rotor period (2 x 50 ms) and recycling delays to 1 s, which corresponds to 1.3*T_1 of the protons. 86 kHz proton decoupling was applied during acquisition of all spectra using the spin64 pulse sequence.[9] 264 (hpdec) or 4096 (CP) transients were summed to obtain the final spectrum.

XAS measurements

XAS measurements, at both Zn K- and In K-edges, were made in fluorescence mode at BM31 of the Swiss-Norwegian beamlines at the ESRF,[10] Grenoble, France, using a Si (111) double

crystal monochromator and a single element drifted Silicon fluorescence detector. The X-ray beam was applied in an unfocused state and shaped to ca. 5 mm (horizontal) and 0.5 mm (vertical) via the uses of slits. The QD samples were measured in solution as sealed capillaries (1 mm o.d., 10 μm wall thickness).

XAS data processing and analysis

The resulting XAS data, were reduced and normalized using the Prestopronto package[11] and or PAXAS.[1] Subsequent analysis of the extracted EXAFS data was performed using EXCURV (v. 9.3).[2]

For the most part, the results reported here derive from the application of single scattering theory, though in some instances full curved wave multiple scattering theory was also used[12,13] to show that, in certain cases (principally from the Zn K-edge, but also in one case from the In K-edge), the derived EXAFS could equally be modelled through taking explicit account of the T_d symmetry of the central atom.

In reporting of the EXAFS analysis, E_F refers to the edge position relative to Vacuum zero (Fermi energy, eV). AFAC is a parameter which account for the proportion of photoelectrons contributing to the EXAFS (0.9 for the Zn K-edge and 1 for the In K-edge), determined through fitting of standards (a Zn foil and zinc oxide, and $InCl_3$ and In_2O_3) measured in transmission. Bond distances are given in Å and the Debye-Waller (DW) factor reported as $2\sigma^2$ (Å^2) where σ is the mean squared displacement of an atom about its equilibrium position.

In assessing the quality of the fits obtained from any tested model, the R-factor (R%) is defined as follows as follows:

$$R\% = \Sigma_i^N \, 1/\sigma_i \, (\chi_i^e (k) - \chi_i^t (k))^2 \times 100\% \qquad (6)$$

Where χ_i^e and χ_i^t are the experimental and theoretical EXAFS respectively and k is the photo-electron wave-vector (Å^{-1}). σ_i is the uncertainty in the data, with $1/\sigma_i = k_i^n/\Sigma j^N \, k_i^n \, (\chi_i^e (k_j))^2$.

The statistical goodness-of fit, the reduced Chi^2 function (x 10^{-6}), is defined as:

$$\chi^2 = 1/(N_{ind} - p)(N_{ind}/N) \, \Sigma_{ind} \, w_i \, (\chi_i^e (k) - \chi_i^t (k))^2 \qquad (7)$$

[1] PAXAS: Program for the analysis of X-ray absorption spectra. N. Binsted, University of Southampton, 1988.
[2] N. Binsted, EXCURV98, CCLRC Daresbury Laboratory computer program, 1998

where N_{ind} is the number of independent data points and p the number of parameters. N_{ind} is determined by the based on the Nyquist theorem:

$$N_{ind} = (2 \times \delta R.\delta k) / \pi \qquad (8)$$

The fitting range used in R space (δR) was 1 – 3 Å, whilst the range of k space used for the fitting of the EXAFS was, depending on the system, between 2.5 – 3 ≤ k ($Å^{-1}$) ≤ 13 – 14.

Figures and Tables

Table S1. Measured concentrations and standard deviations of In, Zn and P of the InP QDs.

		Concentration (mmol/L)		
		In	Zn	P
InP-OLAC	Mean	0.928	-	0.582
	Std. Dev.	0.033	-	0.039
InP:Zn-OLAM	Mean	1.047	0.116	1.078
	Std. Dev.	0.039	0.002	0.068

The purified, as-synthesized InP QDs were measured three times by ICP-MS. The results are summarized in Table S1.

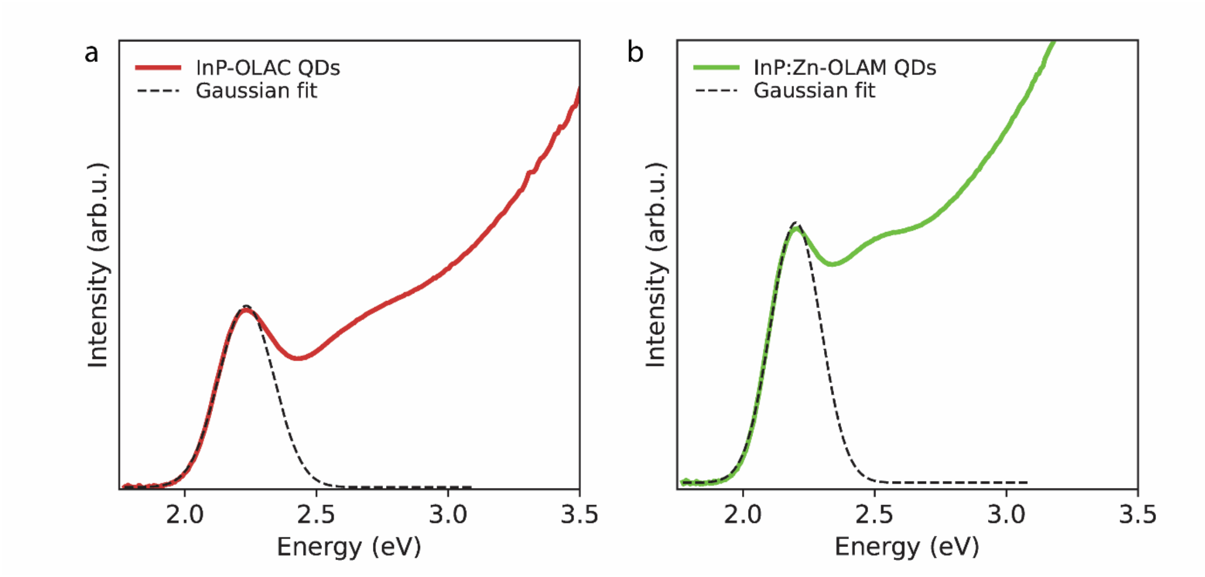

Figure S1. UV-vis absorption spectrum of InP QDs with a gaussian fitting of the first excitonic transition.

Table S2. Detailed fitting parameters of the gaussian fitting of the UV-vis absorption profile.

	x_0 (eV)	A (arb. u.)	σ (eV)	FWHM (meV)	FWHM (nm)
InP-OLAC QDs	2.233	81.22	0.1053	247	62
InP:Zn-OLAM QDs	2.200	117.55	0.0957	225	58

$$g(x) = A \cdot e^{\left(-\frac{(x-x_0)^2}{2\sigma^2}\right)} \qquad (9)$$

$$FWHM = 2\sqrt{2\,ln2}\,\sigma \qquad (10)$$

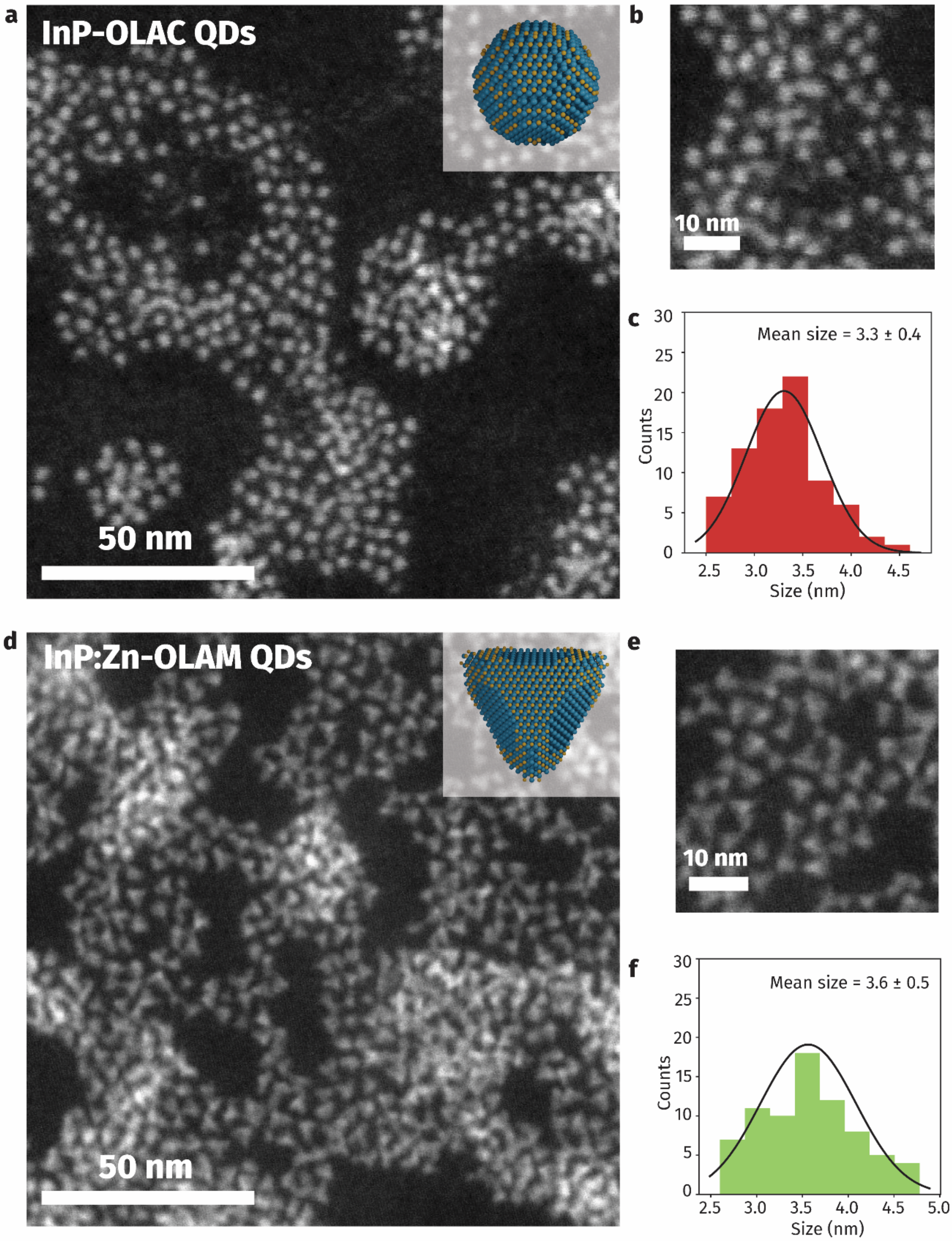

Figure S2. Dark field HAADF-STEM images of as-synthesized (a,b) InP-OLAC QDs and (d,e) InP:Zn-OLAM QDs. Histograms of size distribution derived from measuring the (c) diameter and (f) edge of 75 nanocrystals in the images using ImageJ.

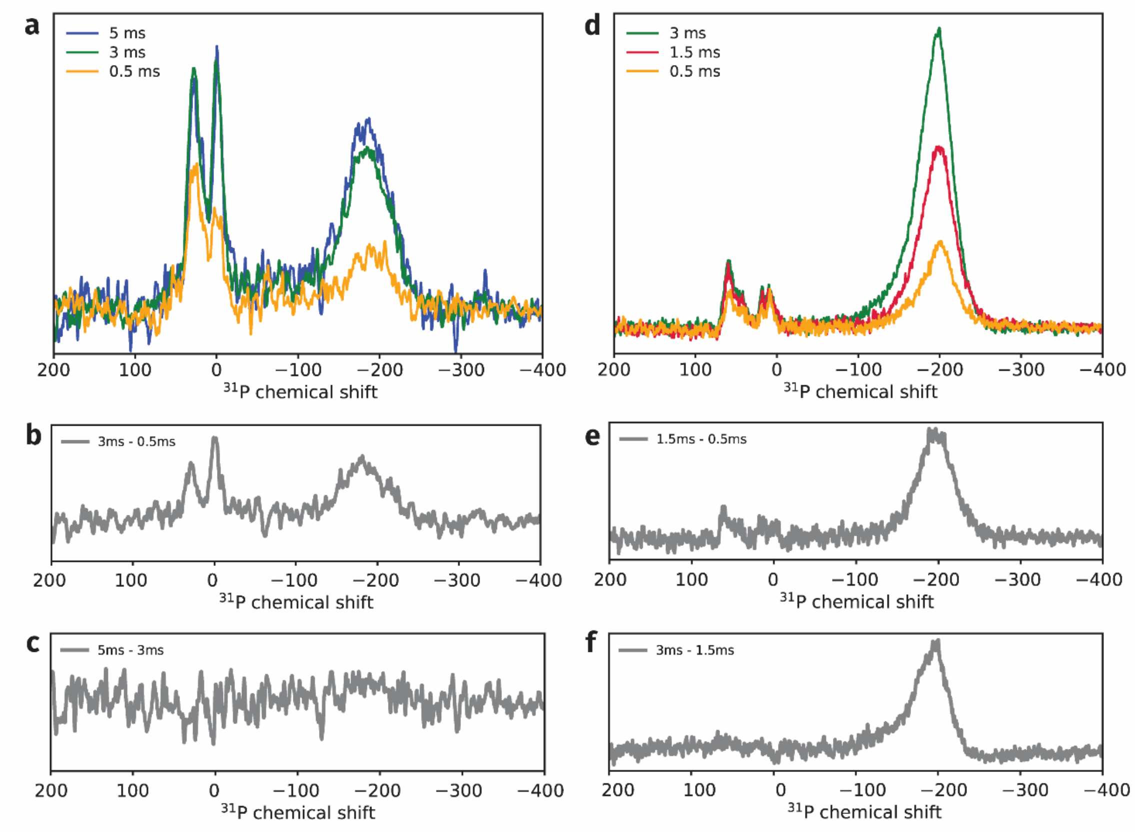

Figure S3. CP ^{1}H-^{31}P MAS NMR spectra of (a-c) InP-OLAC and (d-f) InP:Zn-OLAM QDs for increasing contact times. The increase in the intensity of the signal located at -197 ppm, corresponding to nanoparticulate InP, represents the penetration depth of the signal acquired.

For InP:Zn-OLAM (Figure S3 d-f), the signals located in the 0 – 60 ppm range increase in the intensity from 0.5 ms to 1.5 ms contact time, while there is no further increase from 1.5 ms to 3 ms, as depicted in the plotting of the difference of both spectra (Figure S3 e,f). This result indicates that the presence of phosphorus oxide species is specifically located at the very surface of the nanoparticles.

On the contrary, InP-OLAC shows an increase of both regions from 0.5 ms to 3 ms, and a saturation of the signal obtained from 3 ms to 5 ms. This result indicates that the initial oxidation of the InP-OLAC QDs may penetrate beneath the surface of the QDs.

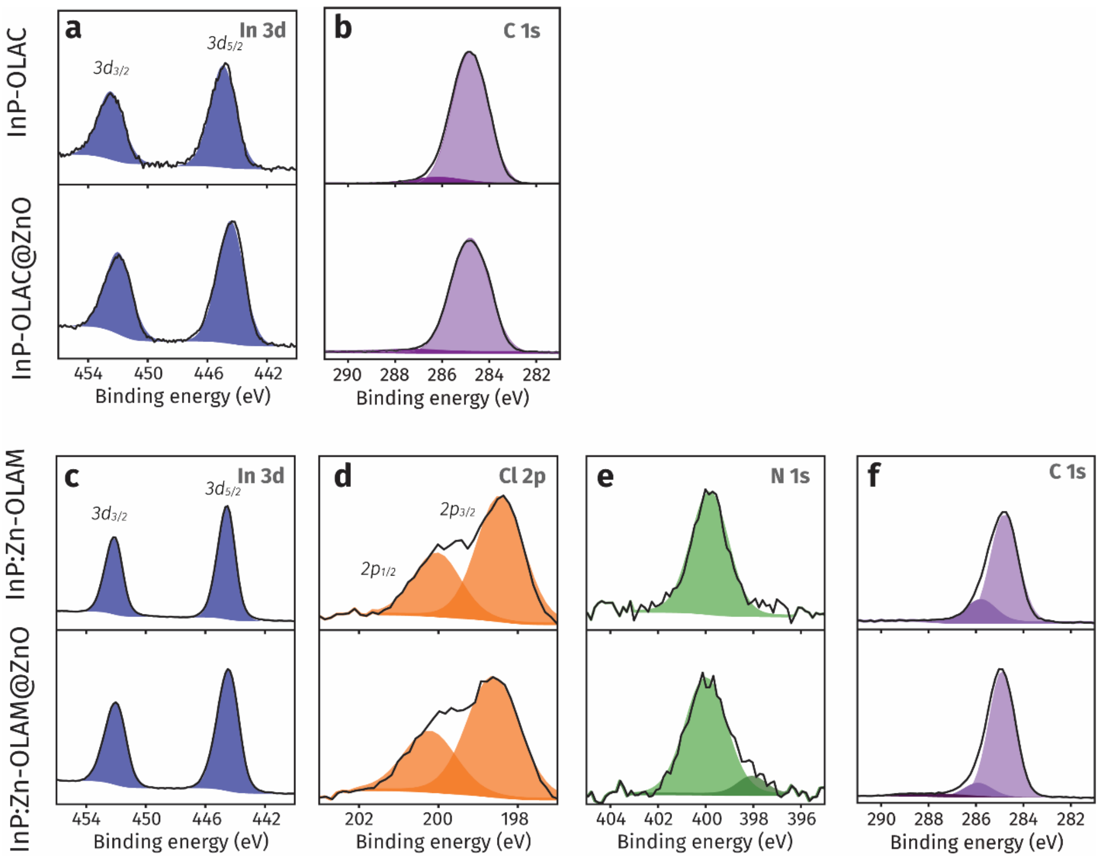

Figure S4. (a-b) XPS spectra of In 3d and C 1s of InP -OLAC QDs (top) and InP-OLAC@ZnO QDs (bottom). (c-f) XPS spectra of In 3d, Cl 2p, N 1s and C 1s of InP:Zn-OLAM QDs (top) and InP:Zn-OLAM@ZnO QDs (bottom).

InP -OLAC

The In 3d region is fitted with one contribution for each spin-orbit peak (separation = 7.54 eV). The position of the C-C bond at core-level C 1s is used to reference the spectra at 284.8 eV. The C1s of as-synthesized InP-OLAC and InP-OLAC@ZnO QDs shows signals corresponding to C-C and O-C=O arising from the presence of oleate ligands.

InP:Zn-OLAM

The In 3d region is fitted with one contribution for each spin-orbit peak (separation = 7.54 eV).

The Cl 2p region is fitted with one contribution for each spin-orbit peak (separation = 1.6 eV). The binding energy corresponds to Cl- at the surface of the QDs “free Cl^-“. After the deposition of the shell, no changes of binding energy are observed.

The N 1s is fitted with one contribution for the InP:Zn-OLAM, corresponding to bound oleylamine. After the shell deposition, a small contribution at lower binding energy emerges which corresponds to free oleylamine.

The position of the C-C bond at core-level C 1s is used to reference the spectra at 284.8 eV. The C1s of as-synthesized InP:Zn-OLAM QDs shows signals corresponding to C-C and C-N arising from the presence of the ligands. After c-ALD, a contribution arising from the O-C=O moiety of OLAC emerges.

Table S3. Detailed XPS peak analysis and peak assignment. Raw data were fit with GL(30) functions with an Iterated Shirley background subtraction (except for C1s, where U 3 Tougaard was used). The position of the C-C bond at core-level C 1s is used to reference the spectra at 284.8 eV. The O 1s region has been excluded as the substrate of the drop casted samples (SiOx@Si) presented the major contribution.

InP-OLAC QDs

	**BE[a] (eV)**	**FWHM (eV)**	**AC[b] (at.%)**	**Description**
In 3d	444.9 ($3d_{5/2}$)	2.03	100%	In-P, In-POx
P 2p	128.6 ($2p_{3/2}$)	1.04	43.3%	P-In
	129.4($2p_{1/2}$)		28.8%	
	132.9($2p_{3/2}$)	2.15	18.6%	[c]P-Ox
	133.8($2p_{1/2}$)		9.3%	
C 1s	284.8	1.79	93.8	C-C
	286.2	2.52	6.2%	O-C=O

[a]BE=binding energy; [b]AC=atomic content.

InP-OLAC@ZnOx QDs

	**BE[a] (eV)**	**FWHM (eV)**	**AC[b] (at.%)**	**Description**
In 3d	444.4 ($3d_{5/2}$)	2.10	100%	In-P, In-POx
P 2p	128.4 ($2p_{3/2}$)	1.44	38.2%	P-In
	129.3 ($2p_{1/2}$)		25.5%	
	132.8 ($2p_{3/2}$)	1.86	304.2%	P-Ox
	133.7 ($2p_{1/2}$)		12.1%	
Zn 2p	1021.9 ($2p_{3/2}$)	2.0	100%	ZnO
C 1s	284.8	1.3	97.4%	C-C
	287.3	3.1	2.6%	O-C=O

[a]BE=binding energy; [b]AC=atomic content.

InP:Zn-OLAM QDs

	BE[a] (eV)	FWHM (eV)	AC[b] (at.%)	Description
In 3d	444.4 ($3d_{5/2}$)	1.41	100%	In-P, In-POx
P 2p	128.7 ($2p_{3/2}$)	1.14	50.1%	P-In
	129.5($2p_{1/2}$)		33.4%	
	132.9($2p_{3/2}$)	1.93	11.0%	[c]P-Ox
	133.8($2p_{1/2}$)		5.5%	
Zn 2p	1021.8 ($2p_{3/2}$)	1.79	100%	Surface doping Zn
Cl 2p	198.4 ($2p_{3/2}$)	1.35	100%	Free Cl^-
C 1s	284.8	1.32	81.5%	C-C
	285.8	1.44	18.4%	C-N
N 1s	399.8	1.79	100%	NH_2

[a]BE=binding energy; [b]AC=atomic content; [c]The higher atomic percentage of POx species seen by XPS in comparison to the quantification with ^{31}P solid-state NMR analysis of the as-synthesized QDs indicates that, although the particle's size is comparable to the escaping depth of the material (3.4 nm and ~2 nm, respectively), XPS remains a surface sensitive technique.

InP:Zn-OLAM@ZnOx QDs

	BE[a] (eV)	FWHM (eV)	AC[b] (at.%)	Description
In 3d	444.0 ($3d_{5/2}$)	1.70	100%	In-P, In-POx
P 2p	128.4 ($2p_{3/2}$)	1.16	32.3%	P-In
	129.3($2p_{1/2}$)		21.5%	
	132.8($2p_{3/2}$)	1.50	30.8%	P-Ox
	133.7($2p_{1/2}$)		15.4%	
Zn 2p	1022.5	2.0	100%	Surface doping Zn, ZnO
Cl 2p	198.4 ($2p_{3/2}$)	1.50	100%	Free Cl^-
C 1s	284.8	1.3	83.7%	C-C
	285.7	1.4	10.5%	C-N
	287.5	3.1	5.8%	O-C=O
N 1s	399.8	1.98	90.6%	NH_2
	397.9	1.36	9.4%	Free NH_2

[a]BE=binding energy; [b]AC=atomic content.

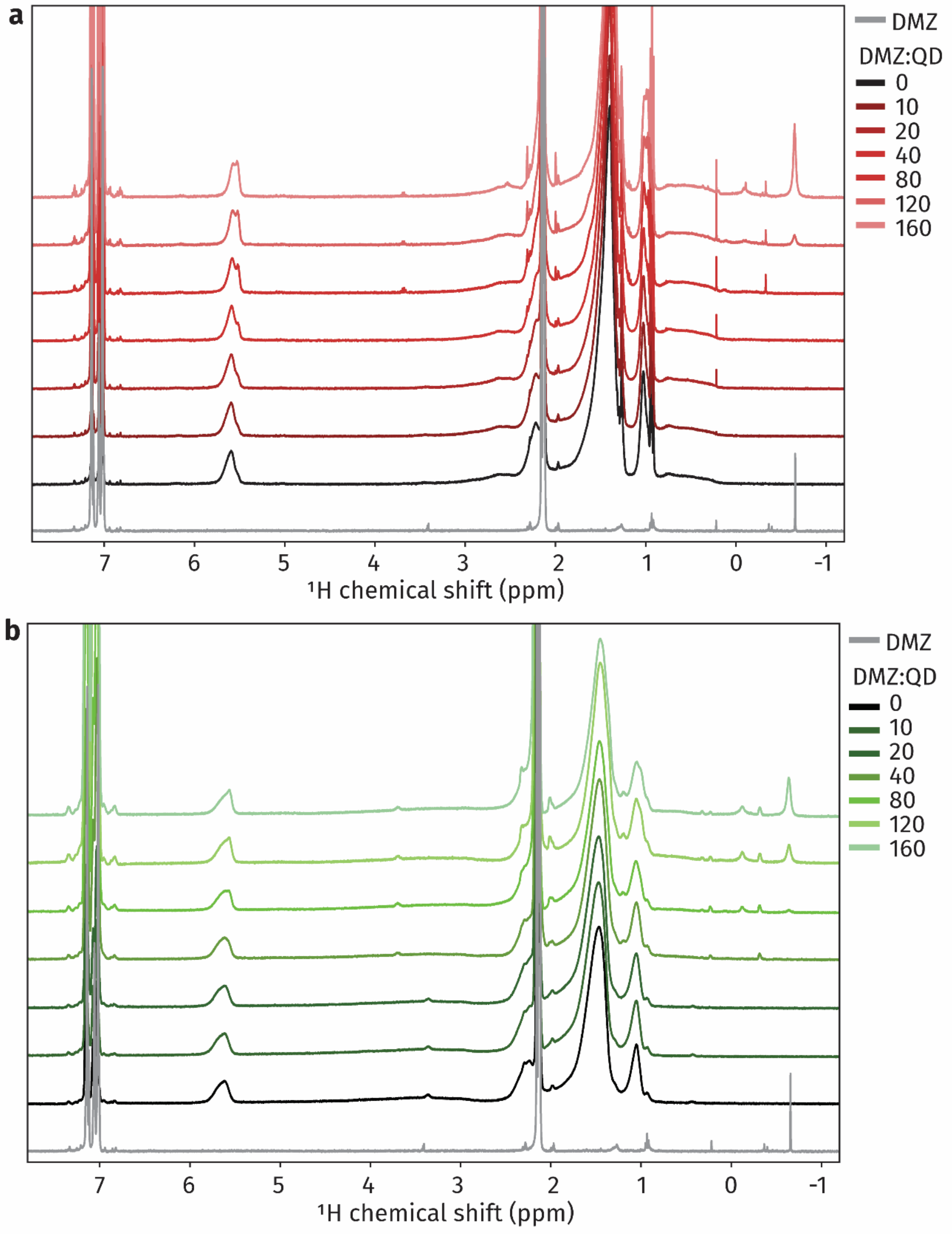

Figure S5. (a,b) ^{1}H NMR of the full spectra of (a) the native oleate ligands and (b) the native oleylamine ligands throughout the addition of DMZ. In grey, the reference spectra of DMZ in toluene-d_8.

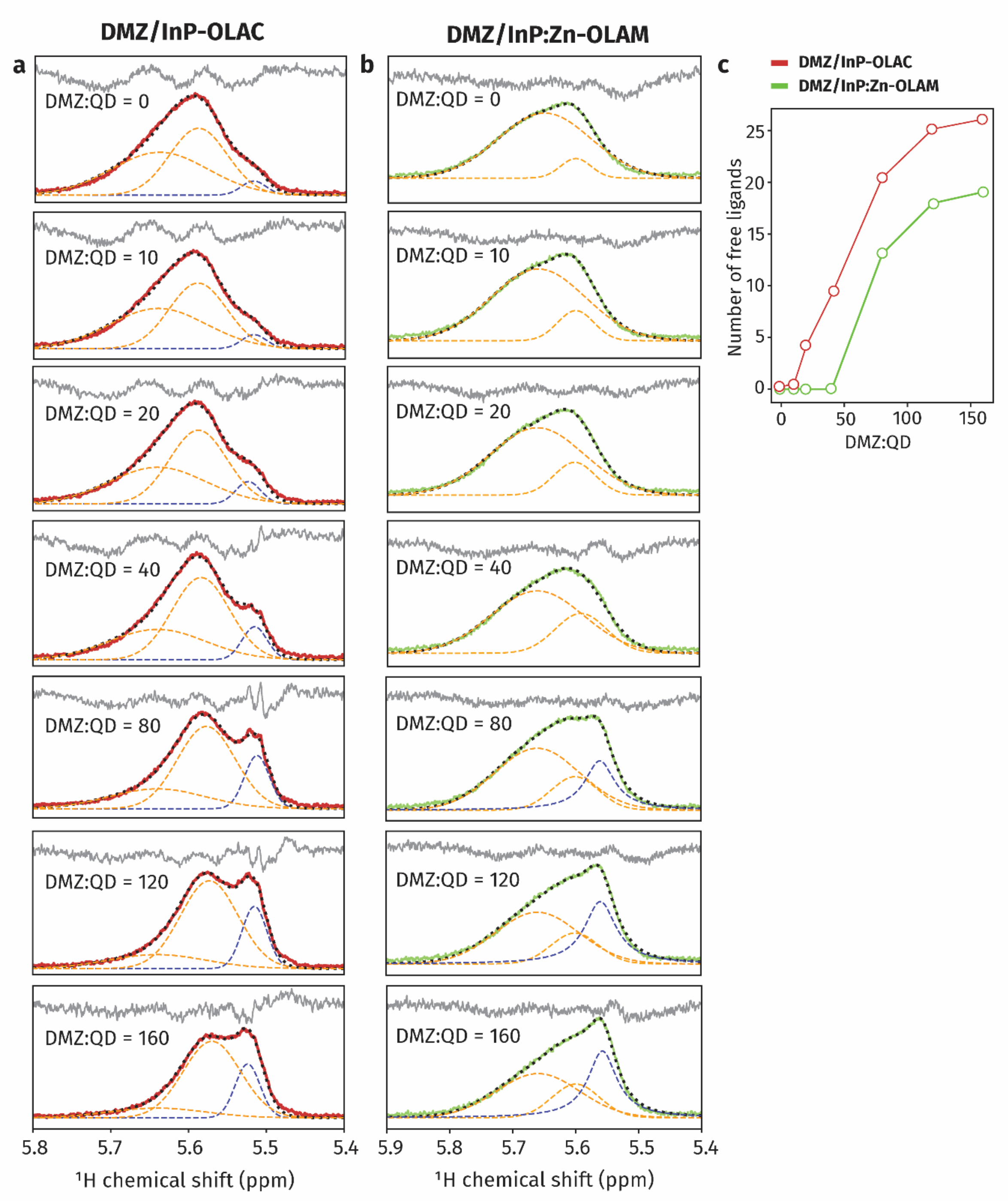

Figure S6. (a,b) Fitting of ^{1}H NMR alkene resonances of (a) oleates and (b) oleylamine through the DMZ titration. Curves in orange correspond to Gaussian functions, and are assigned to tightly bound ligands. Blue curves are Lorentzian functions, and are assigned to free or dynamic ligands. (c) The number of free ligands through the titration calculated from the integration of the Lorentzian curves and the ligand densities calculated by quantitative NMR.

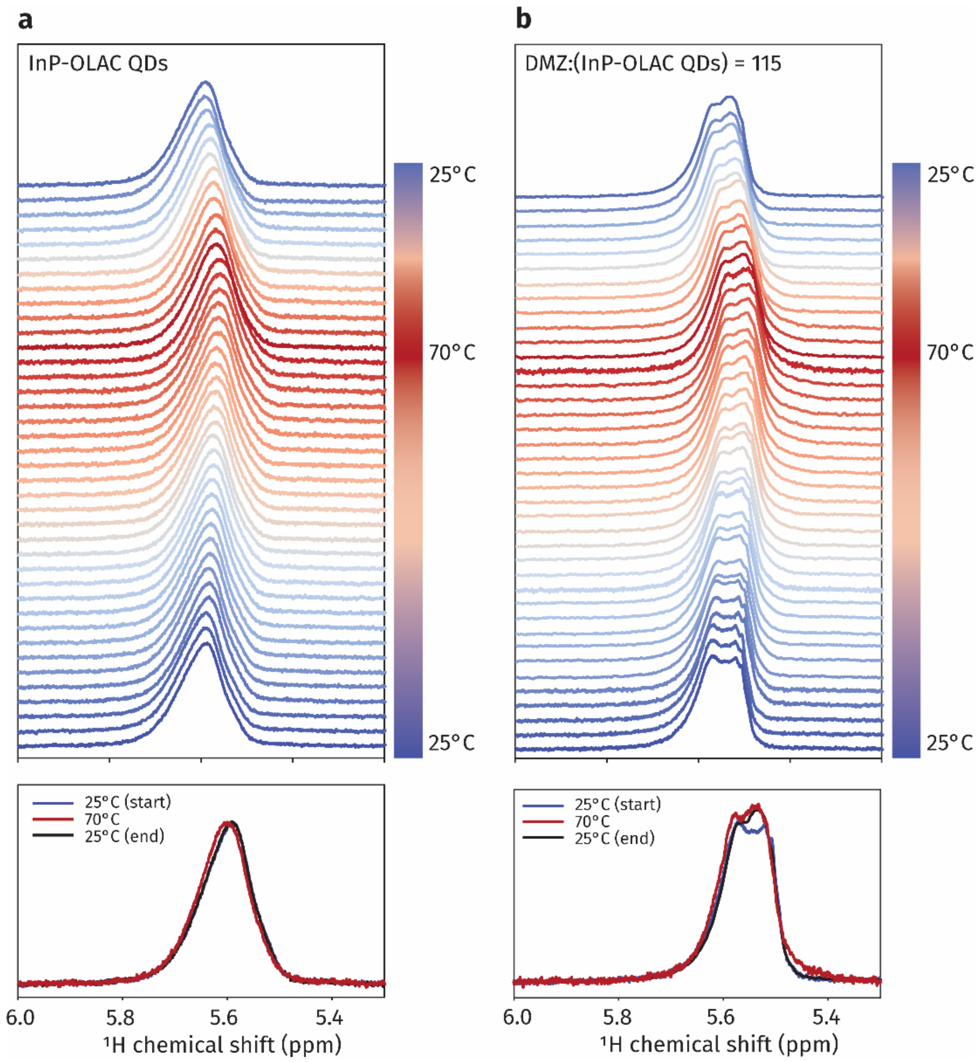

Figure S7. Variable temperature ^{1}H NMR spectra of the alkene region of the as-synthesized InP-OLAC QDs and the same dots after the addition of 115 equivalents of DMZ. Top panels show the alkene signal of oleate ligands through the progressive increase and decrease in temperature. Bottom panels present the spectra at 25ºC before and after the temperature ramp, and the spectra at 70ºC (with the intrinsic upfield shift corrected).

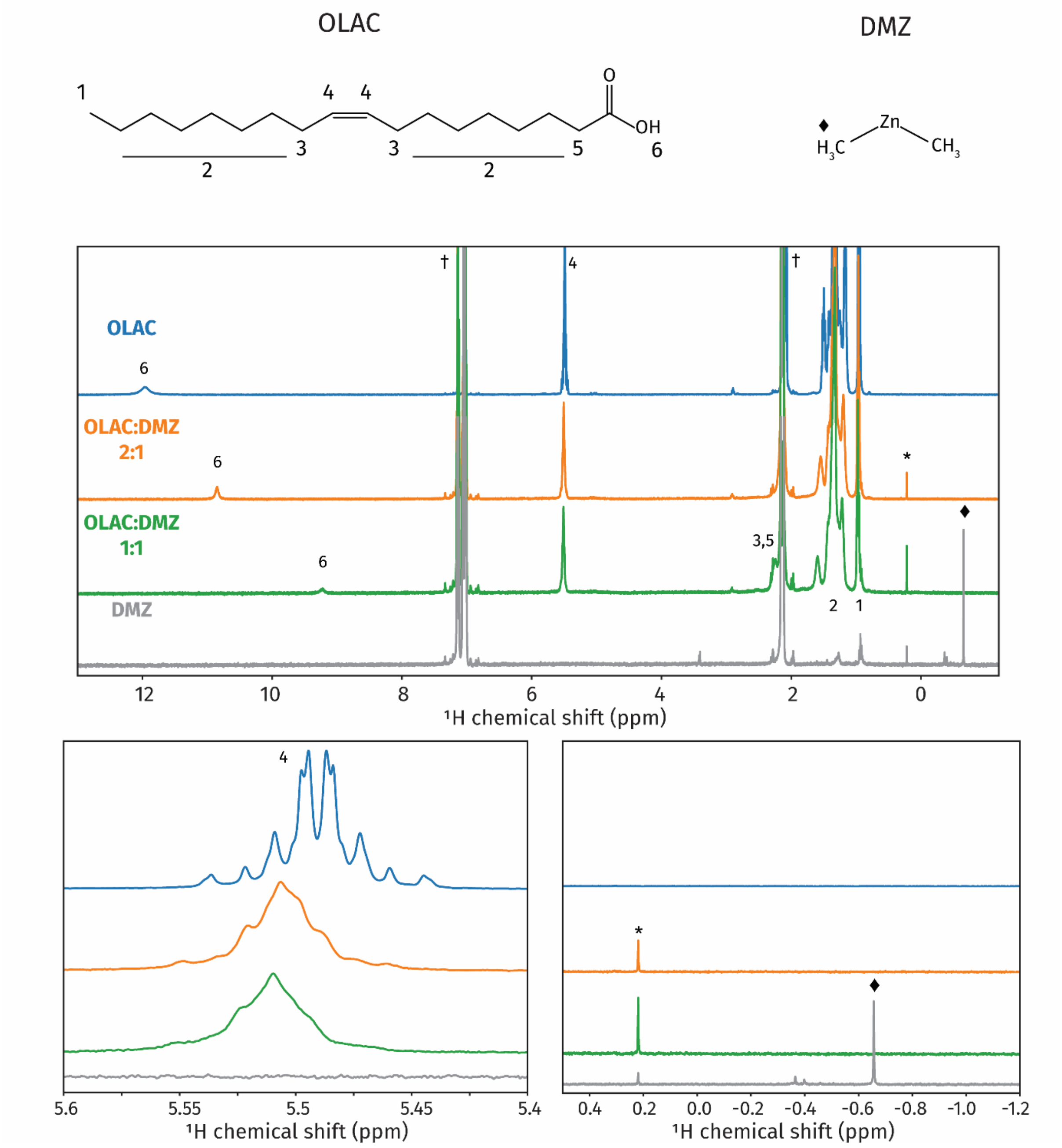

Figure S8. ^{1}H NMR of DMZ, OLAC and a mixture of DMZ:OLAC in 1:1 and 1:2 ratio, performed in toluene-d_8. Along with the different protons of OLAC (labeled with numbers) and DMZ methyl groups (♦), residual solvent resonances can be identified (†), as well as methane (*).

The alkene resonance of the DMZ:OLAC mixtures is broad and does not present the J-coupling typical of OLAC. Additionally, the resonances corresponding to methyl groups of DMZ disappear when reacting with OLAC while the methane resonance intensity increases. These data clearly indicate the formation of a DMZ-OLAC complex through the reaction of the methyl groups of the DMZ with the acidic proton of OLAC.

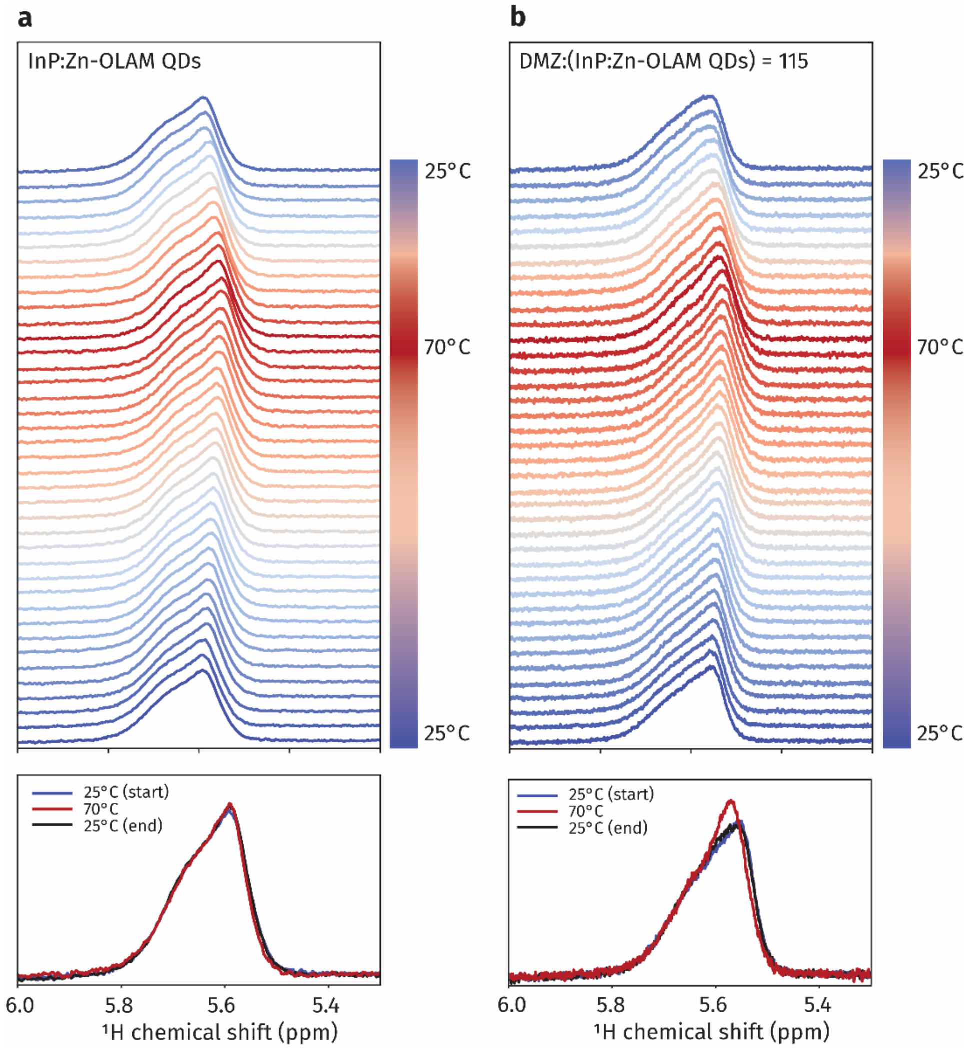

Figure S9. Variable temperature ^{1}H NMR spectra of the alkene region of the as-synthesized InP:Zn-OLAM QDs and the same dots after the addition of 115 equivalents of DMZ. Top panels show the alkene signal of oleylamine ligands through the progressive increase and decrease in temperature. Bottom panels present the spectra at 25ºC before and after the temperature ramp, and the spectra at 70ºC (with the intrinsic upfield shift corrected).

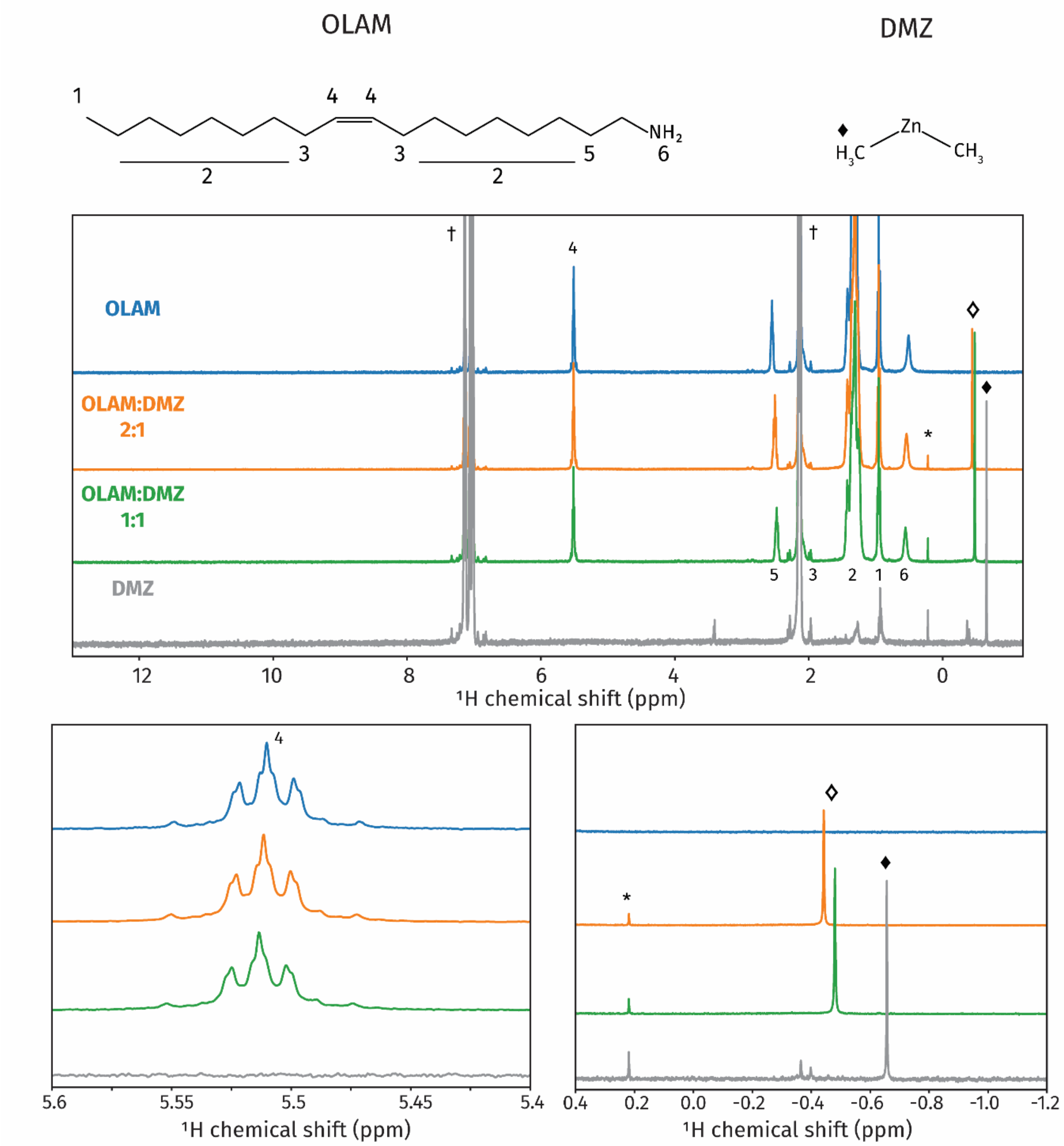

Figure S10. ^{1}H NMR of DMZ, OLAM and a mixture of DMZ:OLAM in 1:1 and 1:2 ratio, performed in toluene-d_8. Along with the different protons of OLAM (labeled with numbers) and DMZ methyl groups (♦), residual solvent resonances can be identified (†), as well as traces of methane (*).

Upon mixing of the DMZ with OLAM, small shifts are observed in resonances 4 and 6, as well as in the chemical shift of the methyl groups (◊). These changes are consistent with the formation of a coordination complex.

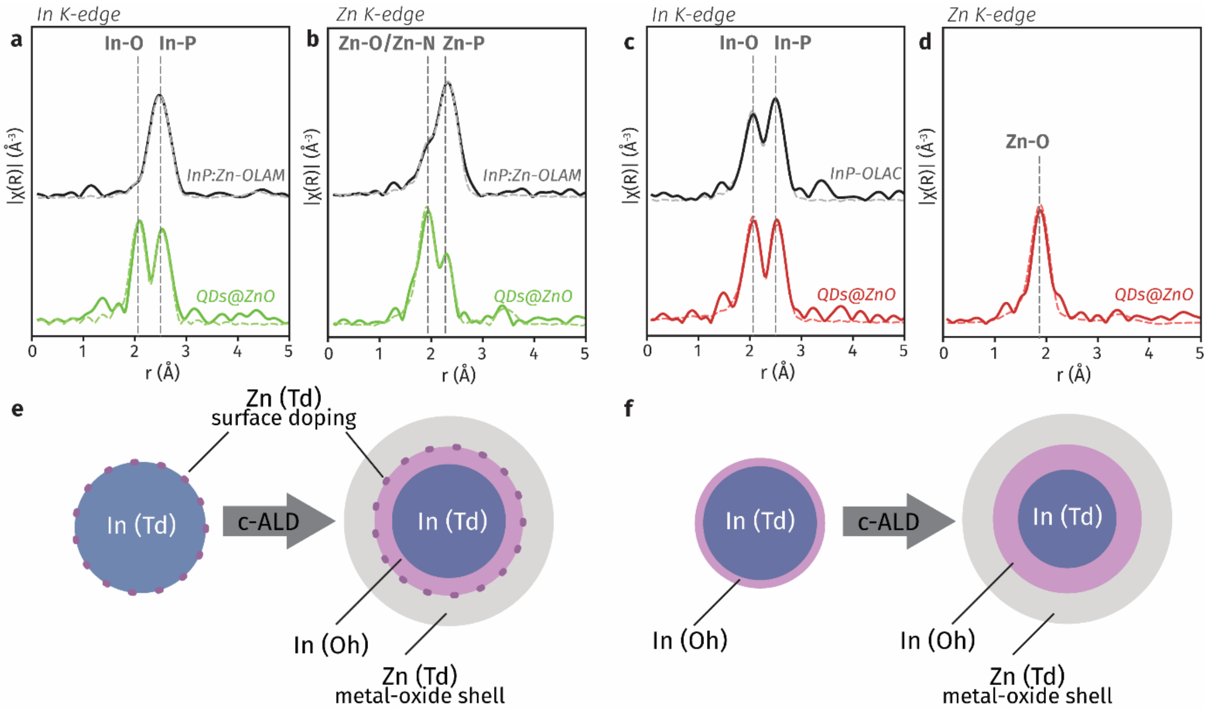

Figure S11. (a-d) XAS analysis including In and Zn K-edge R-space (k^3-weighted) profiles for (a,b) InP:Zn-OLAM QDs and (c,d) InP-OLAC QDs. (e-f) Schematic structural representation of the as-synthesized InP QDs and of the core@shell prepared by c-ALD for (e) the InP:Zn-OLAM system and the (f) InP-OLAC system.

The In K-edge of the as-synthesized InP:Zn-OLAM QDs exhibits an intense peak at 2.52 Å, corresponding to In four-fold coordinated to P (**Table S4**). After the c-ALD process, a shorter interaction appears at 2.12 Å, corresponding to In-O bonding. Interestingly, the average coordination number (CN) around In increases from ~4 to ~5 with the shell deposition, while In remains in a formal In^{3+} oxidation state. The most likely origin of this effect is that the c-ALD process induces surface modifications resulting in a given fraction of the In atoms having a higher coordination than that of bulk InP . Considering this scenario, the fractions of indium that exist in either tetrahedrally (Td) or octahedrally (Oh) shifts from 100% Td for as-synthesized QDs to 45 % Td and 55% Oh for QD@ZnO. Given their size (ca. 3.4 nm), around half of the In atoms are expected to be present at the surface of the QDs. As such, the values of the partitioning between Td and Oh coordination would be consistent with a change of most of the surface In to a six-fold coordination while some retain the native 4-fold coordination and Td geometry along with those in the core.

Moving to the Zn K-edge analysis, the XANES indicates that Zn is present in a Zn^{2+} state before and after c-ALD. For InP:Zn-OLAM QDs, two peaks can be identified in the FT EXAFS (**Table S4**), located at 2.02 and 2.35 Å. Although the first peak could also be fitted with a Zn-O bond, the only marginal amount of oxidation for the as-synthesized samples suggest that this signal is more adequately fitted with a Zn-N bond (CN≈1), arising from the OLAM

coordination at the surface. The second peak can be assigned to Zn-P, with a CN≈3. After the c-ALD process, the sum of the coordination below 3 Å remains 4, within the error, with changes in the average partitioning between Zn-O/Zn-N and Zn-P bonding (**Table S4**). *The fitting of a higher shell to Zn-Zn interactions at 3.3 Å, although weak, is found to be statistically significant and consistent with the development of a ZnO overlayer growth. Notably, we find no indication in our analysis of interstitial or substitutional zinc, indicating that the Zn presence does not go beyond a surface doping for both as-synthesized and core@shell samples.*

In the case of InP-OLAC QDs, the as-synthesized QDs already present peaks corresponding to In-P and In-O, with a total CN of 4.7, at the In K-edge. This can be associated to a partition of 65% Td and 35% Oh for the coordination of In, that arises as a result of the initial oxidation present in these QDs according to ^{31}P NMR and the coordination of oleates to In atoms located at the surface, which is consistent with what reported before.[14] After the deposition of the shell, the total CN increases to 5.5 due to additional In-O interactions, and the fraction of Oh coordinated In increase (75%) at the expense of Td coordinated In (25%), indicating that the change in local bonding symmetry penetrates beyond the outer surface for this case.

After c-ALD we could measure the Zn K-edge for InP-OLAC@ZnO, to find that Zn can be modeled as a ZnO_4 Td center, with no P coordination, and a higher shell with Zn-Zn interactions at 3.3Å.

With this information, we can build up models of the QD structure before and after c-ALD, which are sketched in **Figure S8(e-f)**.

The as-synthesized InP:Zn-OLAM QDs possess a zinc blende crystalline core, constituted of tetrahedrally-coordinated InP, with tetrahedrally-coordinated Zn present as surface doping. Upon deposition of the ZnO shell, approximately half of the In atoms change their coordination from tetrahedral to octahedral due to the formation of an oxidized interface (InPOx), matching a scenario previously predicted in the literature.[15]

The InP-OLAC QDs possess a zinc blende crystalline core, with an outmost layer constituted of a fraction of octahedrally coordinated In, as a result of initial surface oxidation and coordination of oleate ligands. Upon deposition of the ZnO shell, the indium atoms with octahedral coordination are present beyond the outer surface (in the form of InPOx), and the ZnO shell is present with few Zn-P interactions (CN≈0.5).

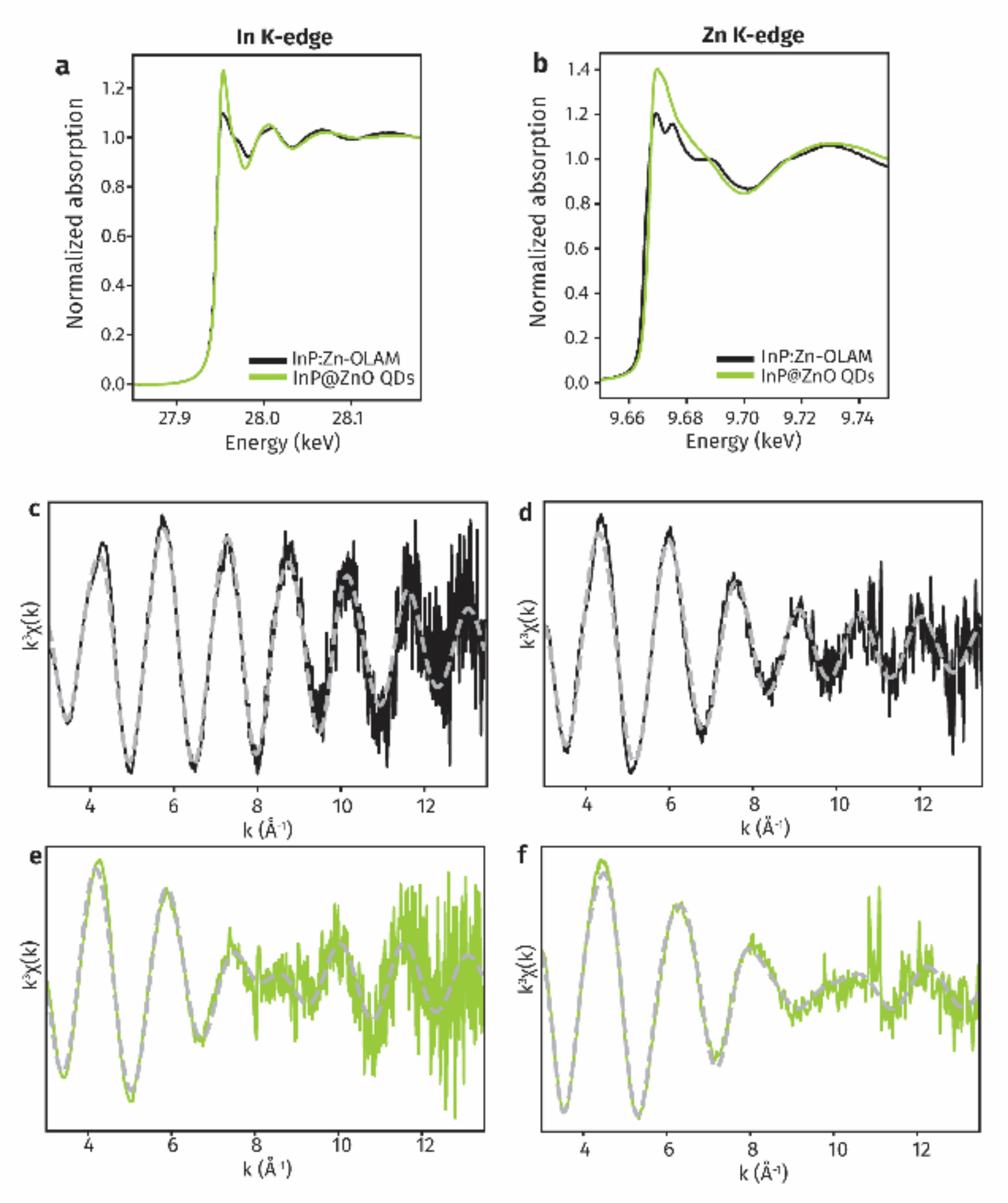

Figure S12. (a) In and (b) Zn K-edge XANES of InP:Zn-OLAM QDs (black) and InP:Zn-OLAM@ZnO QDs (green). k^3-weighted In (c,e) and Zn (d,f) K-edge EXAFS spectra and their respective fittings. InP:Zn-OLAM QDs (black) and InP:Zn-OLAM@ZnO QDs (green).

Table S4. Fitted parameters for EXAFS analyses of the In and Zn edges for the as-synthesized InP:Zn-OLAM QDs and InP:Zn-OLAM @ZnO QDs.

InP:Zn-OLAM QDs

Edge	Bond	C.N.	C.N. (O+P)	R (Å)	DW ($2\sigma^2$) (Å^2)	E_F	R (%)	Chi^2 (x10^{-6})
In	In-P	4.1	4.1	2.52	0.011	-7	31.83	0.64
	In-In	2.1		4.12	0.024			
Zn	Zn-N	1.2	4.3	2.02	0.007	-4.8	29	3
	Zn-P	3.1		2.35	0.013			

InP:Zn-OLAM@ZnO QDs

Edge	Bond	C.N.	C.N. (O+P)	R (Å)	DW ($2\sigma^2$) (Å^2)	E_F	R (%)	Chi^2 (x10^{-6})
In	In-O	2.8	5.1	2.12	0.010	-3	48.3	14.3
	In-P	2.3		2.51	0.013			
Zn	Zn-O	3.0	4.2	1.96	0.013	-3	21.5	0.5
	Zn-P	1.2		2.30	0.012			
	Zn-Zn	1.1		3.31	0.024			

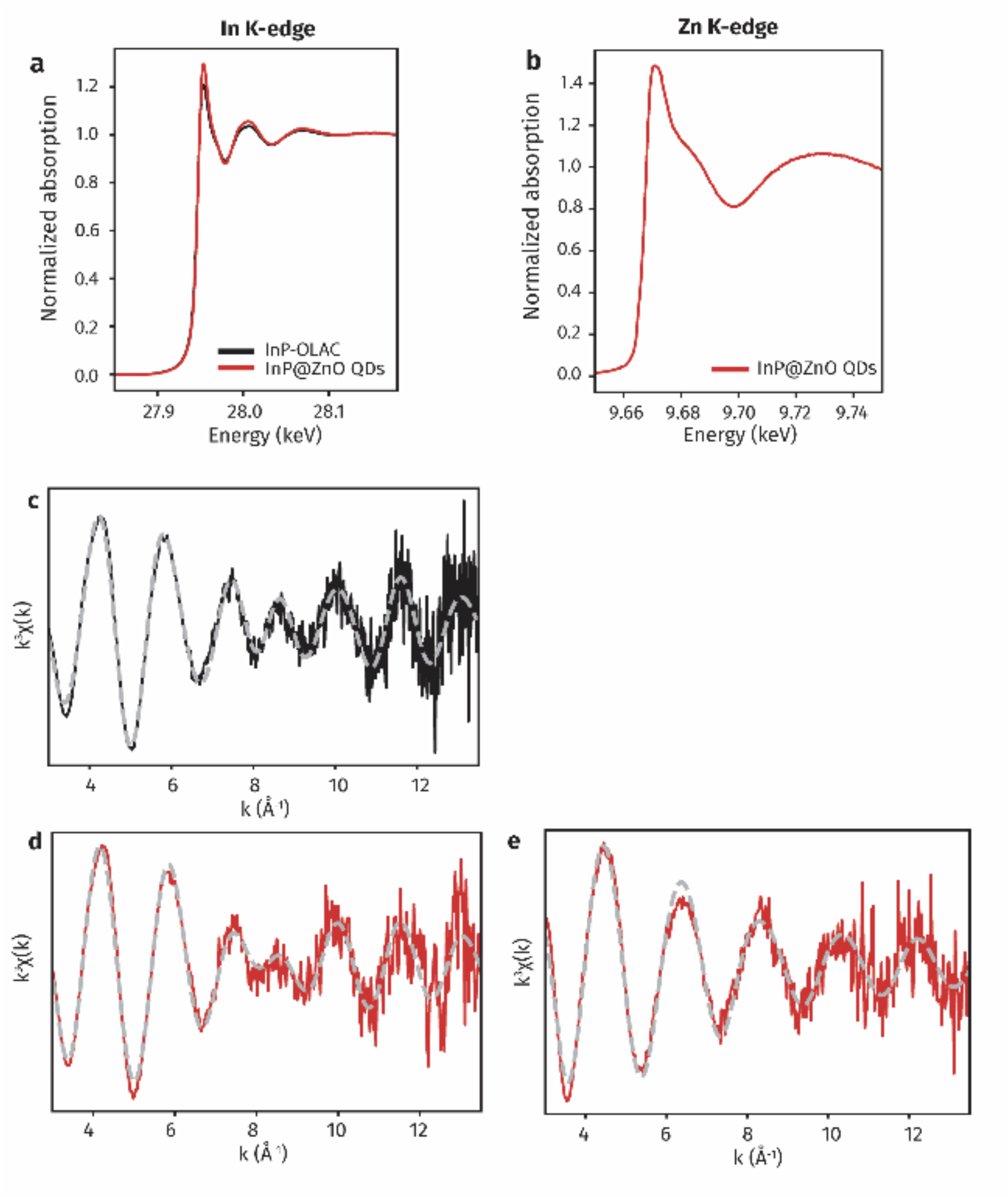

Figure S13. (a) In and (b) Zn K-edge XANES of InP-OLAC QDs (black) and InP -OLAC@ZnO QDs (red). k^3-weighted In (c,e) and Zn (d,f) K-edge EXAFS spectra and their respective fittings. InP -OLAC QDs (black) and InP -OLAC@ZnO QDs (red).

Table S5. Fitted parameters for EXAFS analyses of the In and Zn edges for the as-synthesized InP-OLAC QDs and InP-OLAC@ZnO QDs.

InP-OLAC QDs

Edge	Bond	C.N.	C.N. (O+P)	R (Å)	DW ($2\sigma^2$) (Å^2)	E_F	R (%)	Chi^2 (x10^{-6})
In	In-O	2.7	4.7	2.12	0.009	-4.9	38.4	3.2
	In-P	2.0		2.52	0.014			

InP-OLAC@ZnO QDs

Edge	Bond	C.N.	C.N. (O+P)	R (Å)	DW ($2\sigma^2$) (Å^2)	E_F	R (%)	Chi^2 (x10^{-6})
In	In-O	3.4	5.4	2.12	0.007	-3.5	48.3	14.3
	In-P	2.0		2.51	0.009			
Zn	Zn-O	3.7	4.3	1.95	0.010	-2.9	31.2	0.93
	Zn-P	0.6		2.31	0.012			
	Zn-Zn	1.7		3.30	0.030			

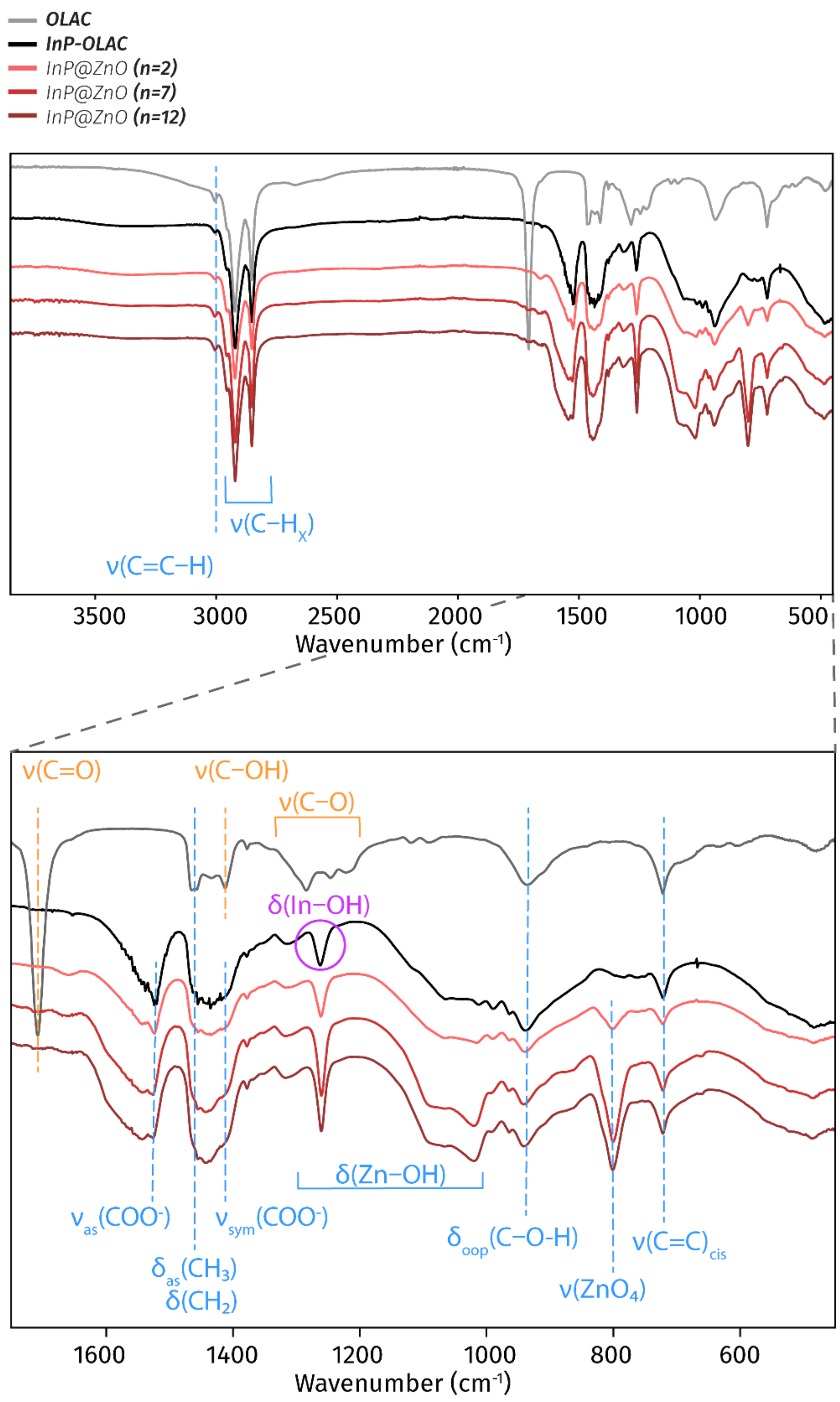

Figure S14. Full FTIR spectra of oleic acid (grey), InP-OLAC QDs (black) and InP@ZnO QDs (shades of red), alongside with a zoom-in of the 1750 – 500 cm^{-1} region. The stretching modes of C=O and C–OH for monodentate oleic acid are highlighted with orange labels. The Δ between these two peaks decreases from ≈300 cm^{-1} to ≈100 – 150 cm^{-1} when OLAC coordinates to the surface of the QDs ($\Delta = \nu_{as}(COO^-) - \nu_{sym}(COO^-)$). The bands at 1258, 1087, 1018 and 800 cm^{-1} are indicative of the presence of the shell. The InP-OLAC QDs show a peak at 1258 cm^{-1} that we ascribe to the presence of intrinsic oxidation of the InP surface.

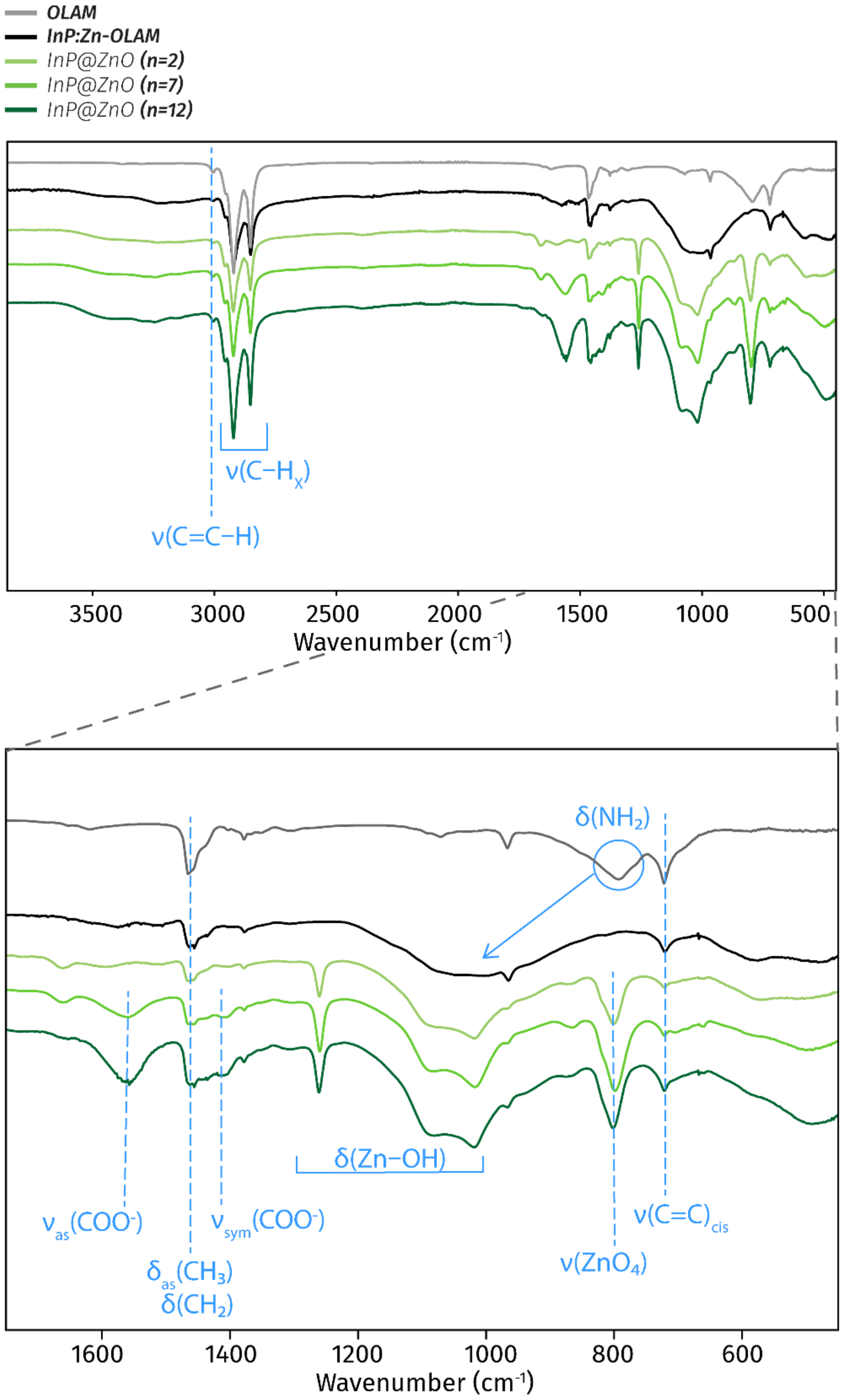

Figure S15. Full FTIR spectra of oleylamine (grey), InP:Zn-OLAM QDs (black) and InP@ZnO QDs (shades of green), alongside with a zoom-in of the 1750 – 500 cm^{-1} region. The symmetric and asymmetric stretching modes of COO^- of oleic acid are present only after the oleic acid addition at n=7, 12. The Δ between these two peaks indicates a major contribution of ligands in a bridging configuration. The bands at 1258, 1087, 1018 and 800 cm^{-1} are indicative of the presence of the shell.

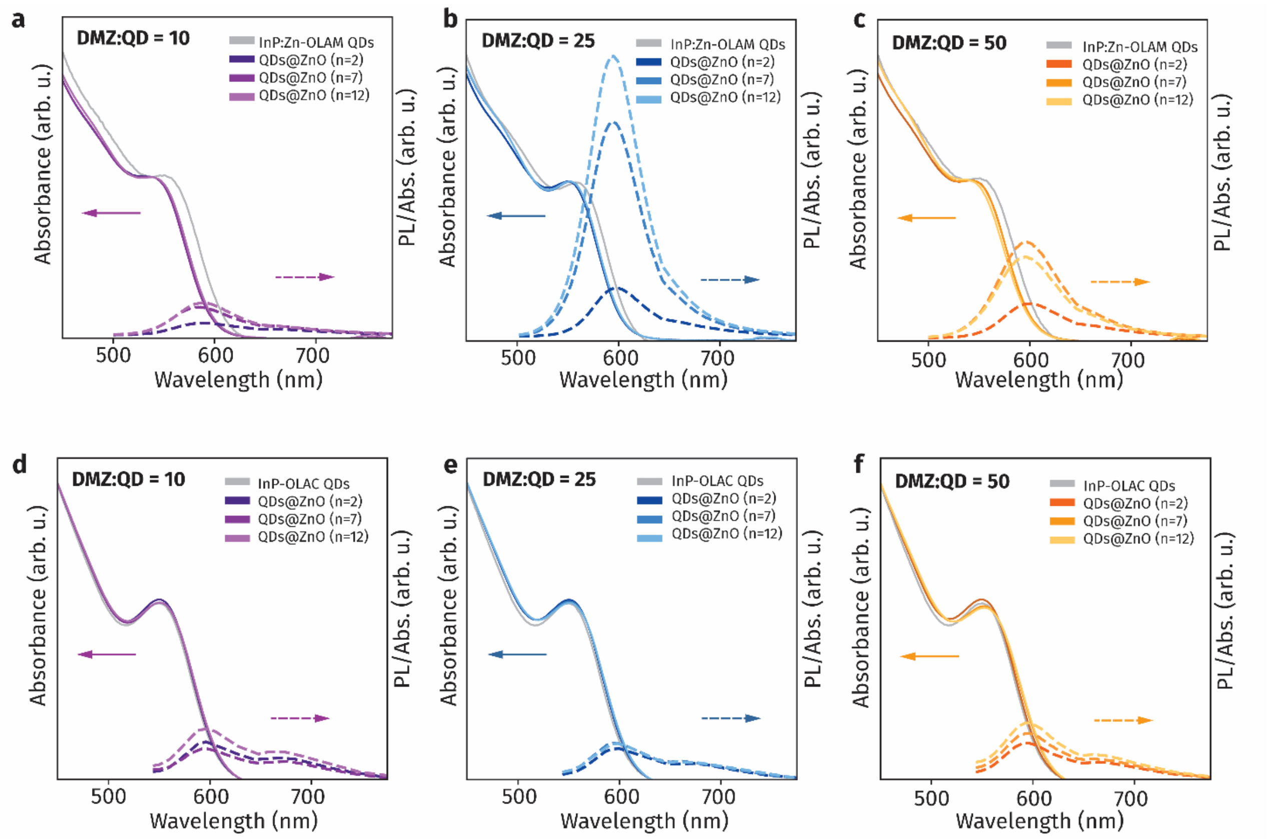

Figure S16. (a-c) For InP:Zn-OLAM QDs, UV-vis spectra and corresponding PL emission of InP QDs after 2, 7 and 12 cycles of c-ALD in which (a) 10, (b) 25 and (c) 50 equivalents of DMZ per quantum dot were added in each cycle. (d-f) Same sample preparation for InP-OLAC QDs. For the sample with the maximal increase in PL emission, n = 12 and DMZ:QD = 25, a PLQY of 2% was measured.

References

(1) Hanrahan, M. P.; Stein, J. L.; Park, N.; Cossairt, B. M.; Rossini, A. J. Elucidating the Location of Cd2+in Post-Synthetically Treated InP Quantum Dots Using Dynamic Nuclear Polarization 31P and 113Cd Solid-State NMR Spectroscopy. *J. Phys. Chem. C* **2021**, *125*, 2956–2965.

(2) Tessier, M. D.; Dupont, D.; De Nolf, K.; De Roo, J.; Hens, Z. Economic and Size-Tunable Synthesis of InP/ZnE (E = S, Se) Colloidal Quantum Dots. *Chem. Mater.* **2015**, *27*, 4893–4898.

(3) Dümbgen, K. C.; Leemans, J.; De Roo, V.; Minjauw, M.; Detavernier, C.; Hens, Z. Surface Chemistry of InP Quantum Dots, Amine-Halide Co-Passivation, and Binding of Z-Type Ligands. *Chem. Mater.* **2023**, *35*, 1037–1046.

(4) Dümbgen, K. C.; Infante, I.; Hens, Z. Localizing Oleylamine Ligands on Amine–Halide Copassivated Indium Phosphide Nanocrystals. *Chem. Mater.* **2023**, *35*, 4393–4403.

(5) Cho, E.; Jang, H.; Lee, J.; Jang, E. Modeling on the Size Dependent Properties of InP Quantum Dots: A Hybrid Functional Study. *Nanotechnology* **2013**, *24*, 1–5.

(6) Xie, L.; Shen, Y.; Franke, D.; Sebastián, V.; Bawendi, M. G.; Jensen, K. F. Characterization of Indium Phosphide Quantum Dot Growth Intermediates Using MALDI-TOF Mass Spectrometry. *J. Am. Chem. Soc.* **2016**, *138*, 13469–13472.

(7) Eichele, K.; Wasylishen, R. E. 31P NMR Study of Powder and Single-Crystal Samples of Ammonium Dihydrogen Phosphate: Effect of Homonuclear Dipolar Coupling. *J. Phys. Chem.* **1994**, *98*, 3108–3113.

(8) Peersen, O. B.; Wu, X.; Kustanovich, I.; Smith, S. O. Variable-Amplitude Cross-Polarization MAS NMR. *J. Magn. Reson. - Ser. A* **1993**, *104*, 334–339.

(9) Fung, B. M.; Khitrin, A. K.; Ermolaev, K. An Improved Broadband Decoupling Sequence for Liquid Crystals and Solids. *J. Magn. Reson.* **2000**, *142*, 97–101.

(10) Van Beek, W.; Safonova, O. V.; Wiker, G.; Emerich, H. SNBL, a Dedicated Beamline for Combined in Situ X-Ray Diffraction, X-Ray Absorption and Raman Scattering Experiments. *Phase Transitions* **2011**, *84*, 726–732.

(11) Figueroa, S. J. A.; Prestipino, C. PrestoPronto: A Code Devoted to Handling Large Data Sets. *J. Phys. Conf. Ser.* **2016**, *712*, 12–16.

(12) Gurman, S. J.; Binsted, N.; Ross, I. A Rapid, Exact Curved-Wave Theory for EXAFS Calculations. *J. Phys. C Solid State Phys.* **1984**, *17*, 143–151.

(13) Gurman, S. J.; Binsted, N.; Ross, I. A Rapid, Exact Curved-Wave Theory for EXAFS Calculations: II. The Multiple-Scattering Contributions. *J. Phys. C Solid State Phys.* **1986**, *19*, 1845–1861.

(14) Gary, D. C.; Flowers, S. E.; Kaminsky, W.; Petrone, A.; Li, X.; Cossairt, B. M. Single-Crystal and Electronic Structure of a 1.3 Nm Indium Phosphide Nanocluster. **2016**, 3–6.

(15) Sluydts, M.; De Nolf, K.; Van Speybroeck, V.; Cottenier, S.; Hens, Z. Ligand Addition Energies and the Stoichiometry of Colloidal Nanocrystals. *ACS Nano* **2016**, *10*, 1462–1474.
